# Identification of Novel Pre-Erythrocytic Malaria Antigen Candidates for Combination Vaccines with Circumsporozoite Protein

**DOI:** 10.1371/journal.pone.0159449

**Published:** 2016-07-19

**Authors:** Cate Speake, Alexander Pichugin, Tejram Sahu, Vlad Malkov, Robert Morrison, Ying Pei, Laure Juompan, Neta Milman, Stasya Zarling, Charles Anderson, Sharon Wong-Madden, Jason Wendler, Andrew Ishizuka, Zachary W. MacMillen, Valentino Garcia, Stefan H. I. Kappe, Urszula Krzych, Patrick E. Duffy

**Affiliations:** 1 Seattle Biomedical Research Institute, Seattle, Washington, United States of America; 2 Department of Cellular Immunology, Malaria Vaccine Branch, Walter Reed Army Institute of Research, Silver Spring, Maryland, United States of America; 3 Laboratory of Malaria Immunology and Vaccinology, National Institute of Allergy and Infectious Diseases, National Institutes of Health, Bethesda, Maryland, United States of America; INSERM, FRANCE

## Abstract

Malaria vaccine development has been hampered by the limited availability of antigens identified through conventional discovery approaches, and improvements are needed to enhance the efficacy of the leading vaccine candidate RTS,S that targets the circumsporozoite protein (CSP) of the infective sporozoite. Here we report a transcriptome-based approach to identify novel pre-erythrocytic vaccine antigens that could potentially be used in combination with CSP. We hypothesized that stage-specific upregulated genes would enrich for protective vaccine targets, and used tiling microarray to identify *P*. *falciparum* genes transcribed at higher levels during liver stage versus sporozoite or blood stages of development. We prepared DNA vaccines for 21 genes using the predicted orthologues in *P*. *yoelii* and *P*. *berghei* and tested their efficacy using different delivery methods against pre-erythrocytic malaria in rodent models. In our primary screen using *P*. *yoelii* in BALB/c mice, we found that 16 antigens significantly reduced liver stage parasite burden. In our confirmatory screen using *P*. *berghei* in C57Bl/6 mice, we confirmed 6 antigens that were protective in both models. Two antigens, when combined with CSP, provided significantly greater protection than CSP alone in both models. Based on the observations reported here, transcriptional patterns of *Plasmodium* genes can be useful in identifying novel pre-erythrocytic antigens that induce protective immunity alone or in combination with CSP.

## Introduction

Malaria is estimated to have caused 854,568 deaths worldwide in 2013 [1], and existing tools for prevention and treatment are losing their efficacy. Nearly five decades after the first demonstration of sterile protection induced by multiple exposures to radiation-attenuated sporozoites (RAS) in mice [2] and humans [3], the world still awaits a licensed and effective malaria vaccine that blocks infection. Manufacturing of RAS for mass-immunization is still in development, while the leading subunit vaccine RTS,S shows 37% efficacy in African children and no significant efficacy in infants against severe malaria [4–6].

RTS,S is based on the *Plasmodium falciparum* (*Pf*) circumsporozoite protein (CSP), the major surface antigen of the sporozoite stage (SS) (6). RTS,S-induced protection from clinical malaria is thought to be mediated by antibodies against the central repeat region together with CD4^+^ T cells [7–9]. Protection against infection correlates most strongly with antibodies induced by RTS,S [10,11], as does sterile immunity induced by the RAS clinical product called *Pf*SPZ Vaccine® that has been tested in humans [12]. Protection induced by RAS in mice [13–15] and in non-human primates [16] depends on CD8^+^ T cells, suggesting that intracellular (intra-hepatocytic) parasite antigens are targets of immunity.

CSP is the immunodominant antigen in RAS vaccines. Although RAS efficacy is significantly reduced in transgenic mice that are tolerized to CSP, increasing the number of doses induces immunity that completely blocks infection in these mice [17–19]. Thus other antigens are likely involved in RAS-induced protection. Human seroreactivity studies similarly suggest that immunity to other antigens expressed by pre-erythrocytic parasites (SS and liver stage (LS)) might enhance CSP-induced protection [20]. No antigens have been shown to enhance the efficacy of RTS,S when used in combination in humans. The thrombospondin-related anonymous protein (TRAP) has been incorporated in a multiantigen virus-vectored platform for heterologous prime boost immunizations (ChAd63-MVA ME-TRAP) which induce partial protection against *Pf* infection [21,22]. Interestingly, RTS,S showed no protective efficacy when combined with recombinant TRAP protein in an AS02 adjuvant formulation [23], emphasizing that some protective immunogens may not be useful in combination with CSP-based vaccines.

Improvements in pre-erythrocytic vaccines have been stymied by our limited knowledge of *Pf* LS antigens. In this paper, we report the identification of protective pre-erythrocytic antigens by transcriptome profiling of *Pf* LS parasites. Candidate antigens were selected from among genes that are over-transcribed in LS compared to either SS or blood stage (BS) parasites. Several of these antigens conferred partial protection against infectious sporozoite challenge in two mouse models: *P*. *berghei* (*Pb*) in C57Bl/6 (B6) and *P*. *yoelii* (*Py*) in BALB/c. When combined with CSP, the sporozoite and liver stage asparagine-rich protein (SLARP) [24], also known as SAP1 [25], and liver-specific protein 1 (LISP1) [26,27] reduced LS burden beyond the reduction achieved with CSP alone in both mouse models. Protection induced by these antigens was mediated by diverse immune mediators including CD8^+^ T cells. We conclude that *Pf* proteins whose genes are over-transcribed during LS development include many protective pre-erythrocytic antigens that might contribute to the development of an effective anti-infection malaria vaccine, including antigens that can be combined with CSP.

## Materials and Methods

### Parasite maintenance

*Pf* (NF54 strain) was transmitted to *Anopheles stephensi* mosquitoes by standard membrane feeding with infected human blood. *Pb* (ANKA strain) and *Py* (17XNL strain) sporozoites were prepared by cyclical transmission in BALB/c mice and *Anopheles stephensi* mosquitoes. Sporozoites were dissected from the salivary glands of mosquitoes on day 21 (*Pb*), day 15 (*Py*) or day 18 (*Pf*) after an infective blood meal, as described previously [28,29]. For challenge studies, sporozoites were counted, adjusted to a given concentration in RPMI 1640 (Life Technologies, Grand Island, NY) with 0.1% normal mouse serum, and used immediately after dissection to ensure maximal infectivity. *Pf* sporozoites were suspended in DMEM/F-12 medium supplemented with 10% FBS, 100 units/ml penicillin and 100 μg/ml streptomycin, and used for axenic culture as described previously [30], or used for *in vitro* infection of HC-04 cells [31].

For microarray analysis, *Pf* parasites were isolated from the blood of Tanzanian children aged between 1 and 4 years [32]. These are the children enrolled in a cohort known as Mother-Offspring Malaria Studies (MOMS), after written informed consent from their mother. Laboratory *Pf* (strain NF54) was cultured *in vitro* in erythrocytes isolated from whole blood as described earlier [33].

### Tiling microarray studies

Custom *Pf* arrays were used to measure the expression profile of genes at different developmental stages, including SS, LS using 24h and 48h axenically cultured parasites, and BS parasite samples collected from Tanzanian children with mild malaria. The array contains 386225 probes, varying in length from 50 to 75 bp (GEO Accession ID: GSE56959), tiling the entire *Pf* genome. RNA samples were sent to Roche-Nimblegen (Roche Nimblegen, Inc., USA) for hybridization and quantification of raw probe intensities. Raw probe intensities were processed with in-house software (R package ‘DuffyMA’). Slides were normalized using Robust Multichip Averaging (RMA) method described in earlier publications [34–36]. Transcript expression was calculated as the geometric mean of all exonic probe intensities for each gene.

### Quantitative real-time PCR (qPCR) verification of *Pf* gene transcription

Total RNA was extracted from the following NF54 strain *Pf* samples: salivary gland sporozoites, infected HC-04 cell samples (24, 48, and 72 h after infection), mixed BS, and uninfected HC-04 cells (negative control). cDNA was prepared from 15 μg of total RNA using the Superscript II RT kit (Invitrogen, Carlsbad, MD) according to manufacturer’s instructions, and qPCR was performed as described earlier [37].

qPCR primer sets were designed using the online program Primer3 [38]. Two hexokinase (PF3D7_0624000) primer sets were used as endogenous controls for data normalization. All primer sequences are available in S1 Table. Data were analyzed according to the relative-standard curve method; standard curves were composed of six 10-fold dilutions of 3D7 genomic DNA.

### Construction of DNA vaccines

The genes listed in Table 1 were amplified by PCR from *Py* (17XNL) and *Pb* (ANKA) genomic DNA or cDNA to produce inserts for ligation-independent cloning. Inserts were then cloned into the mammalian expression plasmid VR1020 (obtained under MTA from Vical Corporation) that was modified to add ligation-independent cloning sites (forward: 5’-CG- CCCAGCGGCACCGGCATG-3’; Reverse: 5’-CACGCACGCGAGCGGGCTTA-3’) using two rounds of site-directed mutagenesis (Stratagene QuikChange II Kit). Because, SLARP, LISP1 and orthologues of PF3D7_0103400 and PF3D7_0304300 are large proteins, multiple fragments were designed, and attempts were made to clone each of them. Of those attempted, we were able to generate 2 out of 6 attempted for *Py*SLARP, 1 of 4 for *Pb*SLARP, 3 of 3 for *Py*LISP1, 2 of 5 for *Pb*LISP1, 1 of 2 each for *Pb*PF3D7_0304300 and *Py*PF3D7_0304300, 1 of 3 for *Py*PF3D7_0103400, and neither of the 2 attempted for *Pb*PF3D7_0103400. Primer sequences for successfully cloned genes are included in S2 Table.

**Table 1:** Pre-erythrocytic antigens selected for vaccine evaluation.

*Pf* Name	*Py* ortholog	*Pb* ortholog	Protein Function	A.A. boundaries in vaccine	
				*Py*	*Pb*
PF3D7_0304600 (CSP)	PY03168	PBANKA_040320	Circumsporozoite (CS) protein (CSP)	35–380	21–320
PF3D7_0730200^a^	PY03000	PBANKA_021430	Adapter-related protein	6–891	5–892
PF3D7_1323000^a^	PY01586	PBANKA_133820	Beta-hydroxyacyl-ACP dehydratase (FabZ)	8–231	1–229
PF3D7_1411500^a^	PY00162	PBANKA_103100	Conserved Plasmodium protein, unknown function	3–980	302–942
PF3D7_1418100 (LISP1)^a^	PY04499	PBANKA_102460	Liver specific protein 1, putative (LISP1)	25–607, 600–1178, and 1172–1732	28–601 and 2579–3254
PF3D7_1207400^a^	PY04162	PBANKA_060590	Conserved Plasmodium protein, unknown function	1–387	153–348
PF3D7_1241500^a^	PY01495	PBANKA_145490	Conserved Plasmodium protein, unknown function	1–467	1–298
PF3D7_1308500^b^	PY04905	PBANKA_140700	Conserved Plasmodium protein, unknown function	14–453	319–456
PF3D7_0727200^b^	PY02096	PBANKA_021130	Cysteine desulfurase, putative (NFS)	3–559	1–541
PF3D7_0818900^b^	PY06158	PBANKA_071190	Heat shock 70 KDa protein, (HSP70)	1–682	1–693
PF3D7_1111200^b^	PY02705	PBANKA_093640	Conserved Plasmodium protein, unknown function	1–360	122–553
PF3D7_1122200^b^	PY05603	PBANKA_092610	Cupin-like protein, putative	63–350	63–447
PF3D7_1134000^b^	PY06981	PBANKA_091440	Heat shock protein 70 (Hsp70-3)	1–663	250–553
PF3D7_1147000 (SLARP)^b^	PY03269	PBANKA_090210	Sporozoite and liver asparagine-rich protein (SLARP)	1356–1989 and 2554–2599	2877–3218
PF3D7_1302200^b^	PY03011	PBANKA_140080	Early transcribed membrane protein 13 (ETRAMP13)	21–215	1–209
PF3D7_1434400^b^	PY03139	PBANKA_101040	Conserved Plasmodium membrane protein, unknown function	1–264	29–261
PF3D7_1456100^b^	PY00669	PBANKA_131980	Serine hydroxymethyltransferase, putative (SHMT)	1–484	5–484
PF3D7_0103400^b^	PY03811	PBANKA_020970	Zinc-carboxypeptidase, putative	623–1203 and 1214–1563	N/A
PF3D7_0304300^b^	PY03529	PBANKA_040290	Conserved Plasmodium protein, unknown function	15–754	1–612
PF3D7_0405500^b^	PY01668	PBANKA_100320	Conserved Plasmodium protein, unknown function	1–198	1–199
PF3D7_0506200^b^	PY00712	PBANKA_110580	Transcription initiation factor TFiid, TATA-binding protein (TBP)	1–277	35–281
PF3D7_1026400^c^	PY07496	PBANKA_051060	Cell division cycle protein 20 homolog, putative (CDC20)	291–552	1–538

^a^Protective in both models

^b^Protective in one model

^c^Protective in neither model

CSP as a positive control.

One Shot TOP10 Chemically Competent *E*. *coli* bacteria (Invitrogen) were transformed with plasmids. Positive clones were selected by DNA sequencing. Sequence-verified clones were used for plasmid DNA preparation for immunization. Plasmid DNA was purified using Geneelute Endotoxin Free Maxiprep kits (Sigma, St. Louis, MO) or Plasmid Endo-Free purification kits (Maxi or Giga, Qiagen, Netherlands) according to manufacturer’s instructions. Plasmids were resuspended at 1 μg/μL in 1X Tris-EDTA buffer (Ambion, Waltham, MA) for Gene Gun (GG) cartridge preparation, at 2 μg/μl in sterile 1X phosphate-buffered saline for intramuscular (IM) injection, and at 0.5 μg/μl in 0.9% NaCl for electroporation (EP) delivery. GG cartridges were prepared as described earlier [39].

### Ethics statement

All procedures were reviewed and approved by Walter Reed Army Institute of Research/Naval Medical Research Center Institutional Animal Care and Use Committee (protocols 12-MVD-31 and 15-MVD-30), National Institutes of Health Institutional Animal Care and Use Committee (protocol LMIV 1E) and Seattle Biomedical Research Center Institutional Animal Care and Use Committee (protocols PD-02 and AO-02) and were performed in a facilities accredited by the Association for Assessment and Accreditation of Laboratory Animal Care International in compliance with the Animal Welfare Act and in accordance with the principles set forth in the “Guide for the Care and Use of Laboratory Animals”, Institute of Laboratory Animals Resources, National Research Council, National Academy Press, 1996. For *Pf* culture, fresh human blood was provided by employee volunteers from SBRI with informed written consent, under the protocol “SBRI Blood Donor Program”, approved by Western IRB.

### Mouse immunizations

Female BALB/c mice, 4–5 weeks old, purchased from Charles River Laboratories. C57Bl/6 (B6) mice and C57Bl/6 × BALB/c F1 mice (CB6F1) (6–8 weeks old) were purchased from The Jackson Laboratories. Mice were maintained under pathogen-free conditions in animal facilities and were fed with autoclaved food *ad libitum*.

For protection studies, B6 mice were immunized with *Pb* DNA vaccines, and BALB/c mice were immunized with *Py* DNA vaccines thrice at 3 week intervals using one of three different routes. Biolistic gold particles coated with plasmid DNA were delivered into shaved mouse abdomens using Helios GG (BioRad, Hercules, CA) at 300 psi of helium gas pressure. Each dose consisted of 2 cartridges at 2.5 μg plasmid DNA/cartridge (total 5 μg plasmid DNA per mouse). For IM injections, 25 μg of plasmid DNA encoding *Py* or *Pb* antigens were mixed with 35 μg of GM-CSF plasmid. EP of 10 μg (*Pb*) or 20 μg (*Py*) of plasmid DNA into anterior tibialis muscle was done using Intramuscular TriGrid Delivery System (Ichor Medical Systems, San Diego, CA) according to manufacturer’s recommendations. Two weeks after the last immunization, B6 mice were challenged with 10,000 *Pb* sporozoites and BALB/c mice were challenged with 20,000 *Py* sporozoites intravenously. Forty to 42 hours after the challenge, livers were harvested for LS burden estimation. For CSP combination immunization studies, mice were immunized three times, 3 weeks apart, by GG for *Pb* and by EP for *Py*. Doses were 2.5 μg plasmid DNA each of individual antigen and CSP for GG, and 10 μg plasmid DNA each for EP. Control mice were immunized with 2.5 μg plasmid DNA of both CSP and empty plasmid vector (EV) control for GG, and with 10 μg plasmid DNA of both for EP. LS burden was estimated after sporozoite challenge by qPCR as described below.

### Evaluation of vaccine efficacy

Total RNA was extracted from liver samples using RNeasy mini kit (Qiagen) (*Py*) or Trizol (*Pb*) according to manufacturer’s instructions. cDNA was synthesized using Superscript III RT kit (Invitrogen) (*Py*) or High-Capacity cDNA Reverse Transcription Kit (Applied Biosystems, Foster City, CA) (*Pb*). Gene expression analysis for *Py* was carried out with 1:40 dilution of cDNA. The qPCR reactions included 1X ABI PowerSYBR (Applied Biosystems) and 0.25 μM of either *Py* 18S rRNA primers (5’-GGGGATTGGTTTTGACGTTTT-3’ and 5’-AAGCATTAAATAAAGCGAATA-3’) or mouse β-actin primers (5’-GGCTGTATTCCCCTCCAT-3’ and 5’-CCAGTTGGTAACAATGCAAT-3’) in a 20 μl volume. qPCR reactions were run on ABI 7500 machine, using the 9600 emulation protocol with annealing/extension step modified to 57.5°C.

*Pb* reaction samples contained the following reagents in 25 μl volume: 12.5 μl of SYBR Green PCR Master Mix (Applied Biosystems), 0.1 μM of either *Pb* 18S rRNA primers (5’-AAGCATTAAATAAAGCGAATACATCCTTAC-3’ and 5’-GGAGATTGGTTTTGACGTTTATGTG-3’) or mouse β-actin gene (5’-GGCTGTATTCCCCTCCAT-3’ and 5’-CCAGTTGGTAACAATGCAAT-3’) and 2 μl of 1:10 dilution of cDNA sample. The reaction was run on 7500 Fast qPCR System (Applied Biosystems) using the following conditions: 15 min at 95°C, 40 cycles with 95°C for 20 s; 60°C for 30 s and 72°C for 50 s.

cDNA standards were prepared as 10-fold serial dilutions of purified PCR product for both 18S rRNA and β-actin from 10^8^ to 10^5^ copies. 18S rRNA was used as an internal control and each reaction was set up in triplicate. Liver of naive mouse served as a negative control. Parasite load was calculated as ratio of 18S rRNA to host β-actin expression. Protection was defined as a statistically significant reduction of parasite burden in the livers of experimental mice compared to mice immunized with EV and computed as described in statistical methods.

### Measurement of CD8 responses to DNA immunization

Two weeks after the final DNA immunization, spleens were harvested from mice and single cell suspensions were prepared from RBC-lysed splenocytes. Cells were stimulated in the presence of brefeldin A with pools of peptides (S3 Table) predicted to have strong binding to H-2K^d/b^, D^d/b^, L^d^ based on the Artificial neural network (ANN) method (www.iedb.org) for 16 hours at 37 °C. Cells were then fixed and permeabilized for intracellular IFN-γ, CD90, and CD8. Stained samples were acquired on a BD LSRII and analyzed using FlowJo v9.6 (Tree Star Inc., Ashland, OR).

### *In vivo* depletion of CD8^+^ T cells

To deplete CD8^+^ T cells, mice were injected intraperitoneally with 100 μg of purified rat anti-mouse-CD8β antibodies (clone 53–6.8, BD Pharmingen, San Diego, CA) 26-28h before the challenge with *Py* sporozoites. Efficiency of depletion was confirmed by surface staining of peripheral blood mononuclear cells (PBMC) prior to the challenge, and of splenocytes at 40h after the challenge, using anti-mouse-CD8α, -CD3 and -CD4 antibodies (see “Flow cytometry”).

### Flow cytometry

The following anti-mouse antibodies from BD Biosciences were used: FITC-conjugated anti-CD3e (clone 145-2C11), PerCP conjugated anti-CD4 (clone GK1.5), and APC-conjugated anti-CD8α (clone 53–6.7). Liver cells and splenocytes were stained according manufacturer recommendations. The cells were washed with PBS + 2% FBS (Hyclone; GE Life Sciences, Logan, UT) and fixed with 4% formaldehyde in PBS. Live/Dead Fixable Dead Cell Stain Kit for UV excitation (Invitrogen) was used to exclude dead cells. Flow cytometry was performed using LSRII system (BD Biosciences, San Jose, CA) and data were analyzed by FlowJo (v. 9.4.10,) software.

### Generation of transgenic parasites

We used a b3D-myc vector for epitope tagging genes of interest [40], to generate the *Py* myc-tagged transgenic lines of *Py*PF3D7_0506200, *Py*PF3D7_1241500, and *Py*PF3D7_0730200. Genes without their stop codons, together with approximately 1.5 kb sequence upstream from the start codon, were amplified by PCR from *Py* 17XNL genomic DNA and inserted upstream of the quadruple myc tag in b3D-myc (list of primers given in S4 Table). The plasmids were subsequently linearized with *Psr* I and integrated into the *Py* 17XNL genome using standard procedures [41]. This integration strategy created two copies of the target gene, both under the control of the endogenous promoter: the original gene and the myc epitope-tagged gene.

### *In vitro* IFA with myc-tagged *Py* LS parasites

Immunofluorescence of myc-tagged LS of *Py* parasite was performed as described earlier [42]. Briefly, HepG2-CD81 cells (5 × 10^4^ Cells/well) were cultured overnight on 8-well chamber slides (LabTek, Rochester, NY) in DMEM medium with 10% FBS at 37°C and 5% CO_2_. The following day, salivary gland sporozoites dissected from mosquitoes were incubated in RPMI with 20% FBS for 30 min at room temperature. Sporozoites (5 × 10^4^) were added to each well of growing HepG2-CD81 cells and incubated for 48h, with media changed every 24h. Infected cells were washed twice with PBS and fixed with formalin, permeabilized, and blocked with 2% BSA in 0.2% Triton X-100 in PBS for 1h at 37°C. Cells were then stained with mouse anti-myc antibody. Alexa-594 or -488-conjugated anti-mouse IgG was used to detect the recombinant protein, rabbit polyclonal antibody to detect UIS4, and DAPI to stain the nucleus. The slides were mounted using FluoroGuard antifade reagent. Fluorescent images were acquired using Olympus 1 X 70 DeltaVision microscope.

### Statistical analysis

Differential gene expression in tiling microarrays was calculated as the ratio of intensity levels between stages, with significance determined by the two sample t-test of the log intensities of all exonic probes. Gene rank percentiles were calculated as a uniform distribution from 0 to 100 based on their ordinal position among all 5536 genes for microarrays, or for the 131 genes assayed for qPCR studies, such that the gene with highest transcription in each sample has a rank percentile of 100.

For meta-analysis of all LS burden studies parasite load was converted to percent reduction for all mice from each study by the equation: $PctReduction\;(Mouse\;i)=\frac{median\;(EV\;group)-Mouse\;i}{median\;(EV\;group)}\times 100\%$, using only the EV mice from the corresponding study. This yields a PctReduction value for all experimental mice and for all control mice, to facilitate combining studies and measuring the statistical significance of each antigen. All groups were compared by Kruskal-Wallis test to determine if differences existed, followed by the non-parametric, 2-sample, 1-sided Mann-Whitney test (R command ‘wilcox.test’) to generate a P-value for individual antigens. The antigen was called protective when the P-value ≤ 0.05 in a comparison of antigen-immunized mice to EV-immunized mice within the same studies. Assays in which none of the antigens including positive control CSP conferred protection were considered uninterpretable and excluded from further analysis. This same strategy was used for both the individual antigen immunization studies and for the CSP combination immunization studies, with the only difference being that the comparator group for the latter studies received EV + CSP combination vaccine.

## Results

### Identification of candidate pre-erythrocytic vaccine antigens

**Fig 1:**
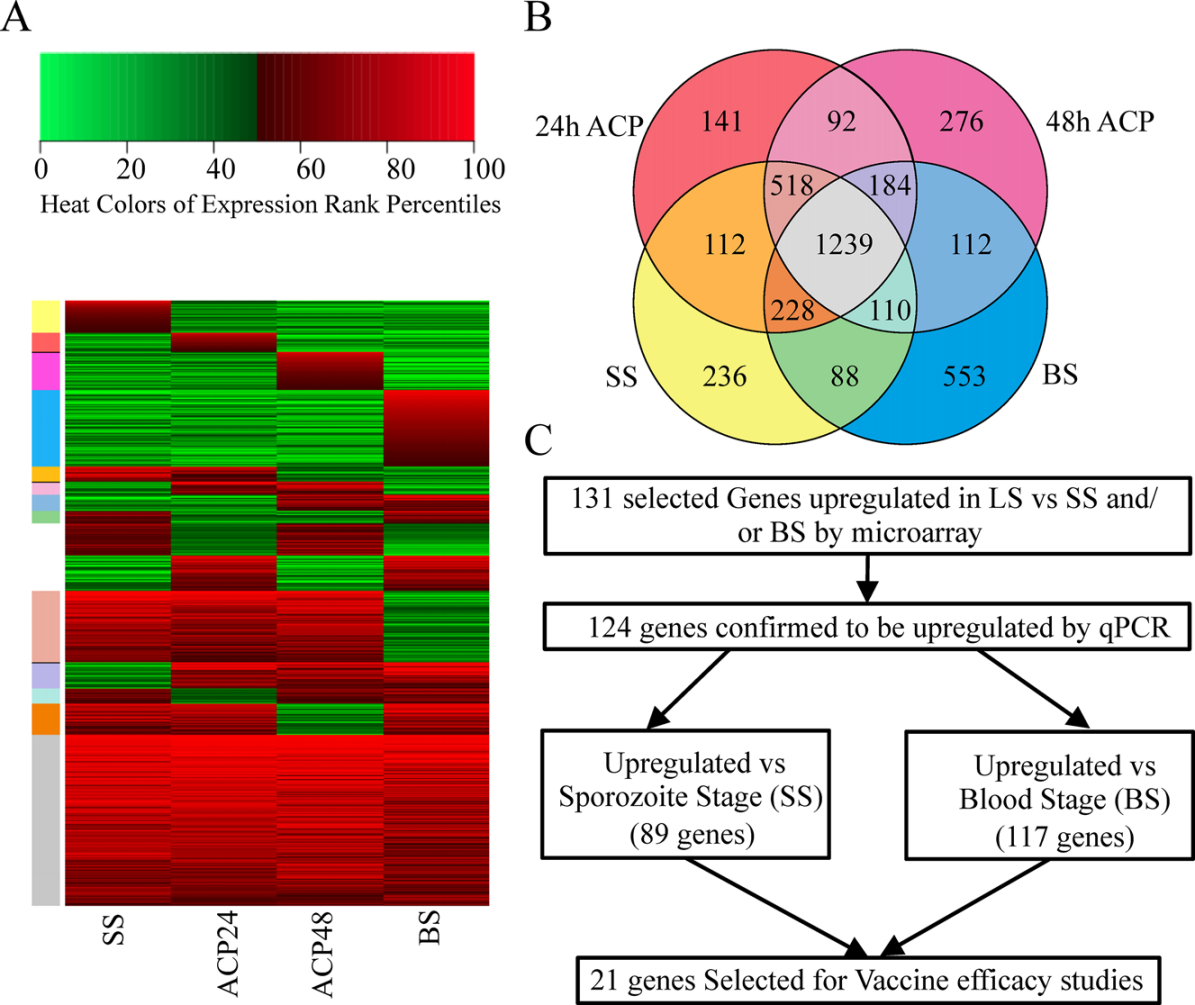
Gene expression profile of *Pf* genes and selection algorithm used for antigen selection. (A) Heat map of gene expression by tiling microarray. Red color represents the genes expressing above the 50^th^ percentile, while green color represents genes expressing below the 50^th^. The heat map shows the expression profile of genes across SS, 24h and 48h axenically cultured LS, and BS parasites. (B) Venn diagram shows stage-specific expression of genes by tiling microarray. Color coding of Venn regions matches the color bar provided for the heat map in Panel A. (C) Selection of genes for vaccine evaluation in two rodent models. One hundred and thirty-one genes were selected from among the upregulated genes identified by tiling-microarray, and 124 were confirmed by qPCR to be transcribed at higher levels in LS versus SS and/or BS parasite samples. Twenty-one of these 124 genes were selected for further evaluation as vaccine candidates.

We cultured NF54 strain *Pf* sporozoites for 24 and 48 hours under axenic conditions [30] to generate material suitable for transcriptional profiling of early *Pf* LS. LS transcriptome data were generated by tiling microarray (Accession No: GSE56959), and we compared the transcriptomes of 24h and 48h LS with those of NF54 strain SS and of BS collected directly from children with mild malaria (Fig 1A and 1B). We identified genes with higher rank percentile transcription at any time point during LS, as compared to either SS or BS, anticipating that this would enrich for genes encoding LS antigens. Using qPCR on parasites infecting HC-04 hepatocytes, we verified that 124 genes among a total of 131 tested (primer sequences listed in S1 Table), were upregulated in LS compared to NF54 strain SS and/or NF54 strain BS transcription. Eighty-nine genes were upregulated during LS compared to SS and 117 were upregulated compared to BS (Fig 1C).

### Evaluation of vaccine efficacy in *Py* and *Pb* models

**Fig 2:**
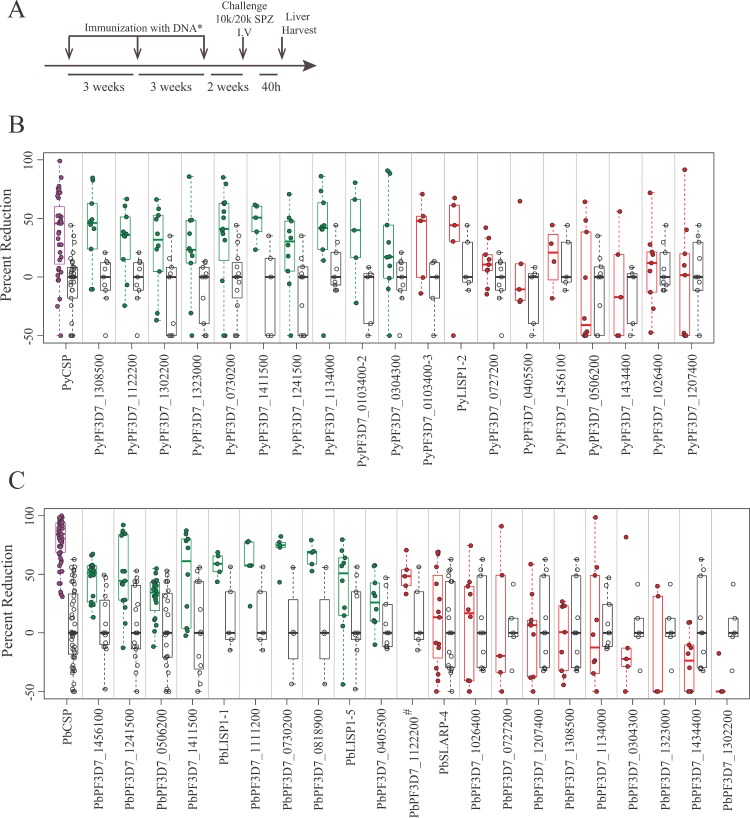
GG DNA immunization and reduction of LS parasite burden post-sporozoite challenge. (A) Experimental design for the immunization and challenge studies. Mice were immunized 3 times at 3 week intervals with VR1020 plasmid DNA carrying the *Pb* or *Py* antigen. Two weeks after the last boost mice were challenged with 10,000 *Pb* or 20,000 *Py* sporozoites intravenously and livers were harvested 40h post-challenge. *DNA dose is 5 μg (GG), 25 μg + 35 μg GM-CSF DNA (IM) or 20 μg (EP). (B) Meta-analyses of 7 independent immunization experiments and resulting LS parasite burden reduction in *Py* in BALB/c model by GG immunizations. (C) Meta-analyses of 10 independent immunization experiments and resulting LS parasite burden reduction in *Pb* in C57Bl/6 model induced by GG immunizations. Each circle represents one mouse. Green color indicates significant difference as compared to EV immunized groups tested in the same immunization studies (p<0.05). Red color indicates p>0.05 and therefore no significant difference in LS parasite burden reduction as compared to EV immunized group. Purple color indicates LS parasite burden reduction by CSP (positive control). A complete statistical analysis is provided in S5 Table.

Twenty-one genes upregulated in *Pf* LS parasites and with predicted orthologues in *Py* and *Pb* were selected to conduct immunization/challenge studies in two rodent models used for initial and confirmatory screening (*Py* in BALB/c and *Pb* in B6 mice, respectively) (Fig 1C and Table 1). The selected candidates included genes with or without predicted export sequences (e.g., canonical eukaryotic signal peptide (SP) or transmembrane (TM) domains, or parasite-specific PEXEL/VTS motifs [43,44]), and with varying patterns of transcription across SS, LS and BS parasites (Table 2). CSP DNA vaccine was used as a positive control and benchmark of partial protection [45], and EV served as a negative control. Mice were immunized 3 times with DNA constructs, and then challenged with homologous sporozoite species (Fig 2A); protection conferred by each DNA vaccine was measured as reduction of LS parasite burden (18S rRNA level quantified by qPCR) compared to EV control tested in the same immunization studies (Fig 2B and 2C). Using GG immunization, 10/21 novel antigens significantly (P<0.05) reduced LS burden compared to the EV immunized group in the *Py* model (Fig 2B and S5 Table) as did 9/21 in the *Pb* model (Fig 2C and S5 Table), with 3 antigens (orthologues of PF3D7_0730200, PF3D7_1241500 and PF3D7_1411500) demonstrating protection in both models.

**Table 2:** Protein features and transcriptional profiles of selected candidate genes.

*Pf* Gene Name	Protein Feature				Upregulated transcription in LS vs	
	Protein Length	TM	SP	PEXEL/VTS	BS	SS
PF3D7_0304600 (CSP)	397	1	1	2	Yes	No
PF3D7_0730200^a^	858	0	0	0	Yes	Yes
PF3D7_1323000^a^	230	1	1	0	Yes	Yes
PF3D7_1411500^a^	947	0	0	0	Yes	Yes
PF3D7_1418100 (LISP1)^a^	3597	0	0	0	Yes	Yes
PF3D7_1207400^a^	395	0	0	0	Yes	No
PF3D7_1241500^a^	420	0	0	0	Yes	Yes
PF3D7_1308500^b^	359	0	0	0	Yes	No
PF3D7_0727200^b^	553	0	0	1	Yes	No
PF3D7_0818900^b^	677	0	0	1	Yes	Yes
PF3D7_1111200^b^	594	0	0	0	Yes	Yes
PF3D7_1122200^b^	446	0	0	0	Yes	Yes
PF3D7_1134000^b^	663	0	0	0	Yes	Yes
PF3D7_1147000 (SLARP)^b^	2940	0	0	0	Yes	No
PF3D7_1302200^b^	229	2	1	0	Yes	No
PF3D7_1434400^b^	285	1	0	0	Yes	No
PF3D7_1456100^b^	462	0	0	0	Yes	Yes
PF3D7_0103400^b^	1620	0	0	0	Yes	No
PF3D7_0304300^b^	1429	0	0	0	Yes	Yes
PF3D7_0405500^b^	224	0	0	1	Yes	Yes
PF3D7_0506200^b^	327	0	0	0	Yes	Yes
PF3D7_1026400^c^	603	0	0	0	Yes	No

^a^Protective in both models

^b^Protective in one model

^c^Protective in neither model

CSP as a positive control.

Protective efficacy of antigens can vary depending on the route of immunization [45]. Therefore, in addition to GG immunization, we tested many of the *Py* and *Pb* immunogens using EP, and in addition IM injection for many *Py* immunogens. Most (6/10) DNA vaccines that protected by GG immunization for *Py* also conferred functional immunity when delivered by IM and/or EP routes; 4/9 *Pb* immunogens that conferred protection by GG also conferred protection by EP administration (S1 Fig). In some cases, protection was only seen with one route of administration: for example, *Py* antigens *Py*PF3D7_1026400and *Py*PF3D7_1207400 only achieved significant protection by either EP or IM delivery respectively. Notably, the sample size was not uniform across antigens, in part because some experiments were uninterpretable; for this reason the power to detect differences varied across antigens, and therefore some differences of relatively smaller magnitude were significant while those of greater magnitude did not achieve significance. GG immunization has been thought to confer greater protective efficacy [46] in comparison to the IM route, but IM immunization may achieve superior results in a subset of antigens [45]. For unclear reasons, IM administration yielded poor protective activity in the *Pb* model and was hence discontinued after testing only a minority of the antigens (data not shown). In total, 6 antigens (orthologues of PF3D7_1411500, PF3D7_0730200, PF3D7_1241500, PF3D7_1323000, PF3D7_1207400 and LISP1) significantly reduced LS burden after sporozoite challenge in both *Py* and *Pb* mouse models of malaria, by at least one route of administration (Fig 2, S1 Fig and S5 Table). The observation that most antigens conferred protection with more than one delivery platform, and also that they conferred protection in more than one mouse model, suggest that these are robust targets for further vaccine development.

### Protection induced by novel LS antigens is mediated by diverse mechanisms

Previous studies in rodents demonstrated that protective immunity induced by vaccination with RAS is mediated by multi-factorial mechanisms including neutralizing antibodies that block sporozoite invasion and effector CD8^+^ T cells that eliminate infected hepatocytes. To investigate if CD8^+^ T cells consistently contribute to protection induced by novel LS antigens, we confirmed that 4 antigens conferred protection in CB6F1 hybrid mice, an additional mouse model which we surmised might yield more diverse immune responses owing to its more diverse MHC haplotypes. Three of these antigens (*Py*PF3D7_1456100, *Py*SLARP, *Py*PF3D7_1207400) elicited IFN-γ responses from CD8^+^ T cells, while *Py*PF3D7_0506200 apparently did not (S2A Fig), based on stimulation with a pool of peptides predicted to be CD8^+^ T cell epitopes for H-2K^d/b^, D^d/b^, L^d^ by the Artificial neural network (ANN) method (www.iedb.org). CB6F1 mice immunized by GG with these *Py* DNA antigens, as well as benchmark antigen *Py*CSP, received anti-CD8β antibodies to deplete CD8^+^ T cells 24 hours before the challenge with *Py* sporozoites (S2B Fig). We had previously determined that a single i.p. injection with 100μg of anti-CD8 monoclonal antibodies one day prior to challenge eliminated >80% of CD8^+^ T cells in peripheral blood and spleen (S3 Fig) and that administration of RatIg control antibody had no effect on CD8^+^ T cells (data not shown). CD8 depletion completely abrogated protection induced by novel LS Ags *Py*PF3D7_1456100 and *Py*SLARP in CB6F1 mice (S2B Fig). Protection induced by *Py*PF3D7_1207400 was partially mediated by CD8^+^ T cells–reduction of LS parasite burden dropped from 31% to only 13% after depletion. Depletion of CD8^+^ T cells did not cause any loss of protection in mice immunized with *Py*PF3D7_0506200, the antigen for which we could not detect CD8^+^ T cell responses after DNA vaccination. Notably, CSP induced protection did not require CD8^+^ T cells in this model, despite the fact that the CSP immunogen elicited peptide-specific CD8^+^ T cell responses. Our data suggest that protection induced by novel pre-erythrocytic antigens can be mediated by diverse mechanisms that vary by antigen, some of which include induction of effector CD8^+^ T cells.

### Efficacy of combination vaccines with CSP and novel antigens

The leading human malaria vaccine is based on the antigen CSP. CSP reduces LS burden and induces partial protection when delivered as a DNA vaccine in the *Pb* and *Py* models [45,47]. We assessed whether the addition of our novel antigens might provide greater protection than that achieved with CSP alone.

**Fig 3:**
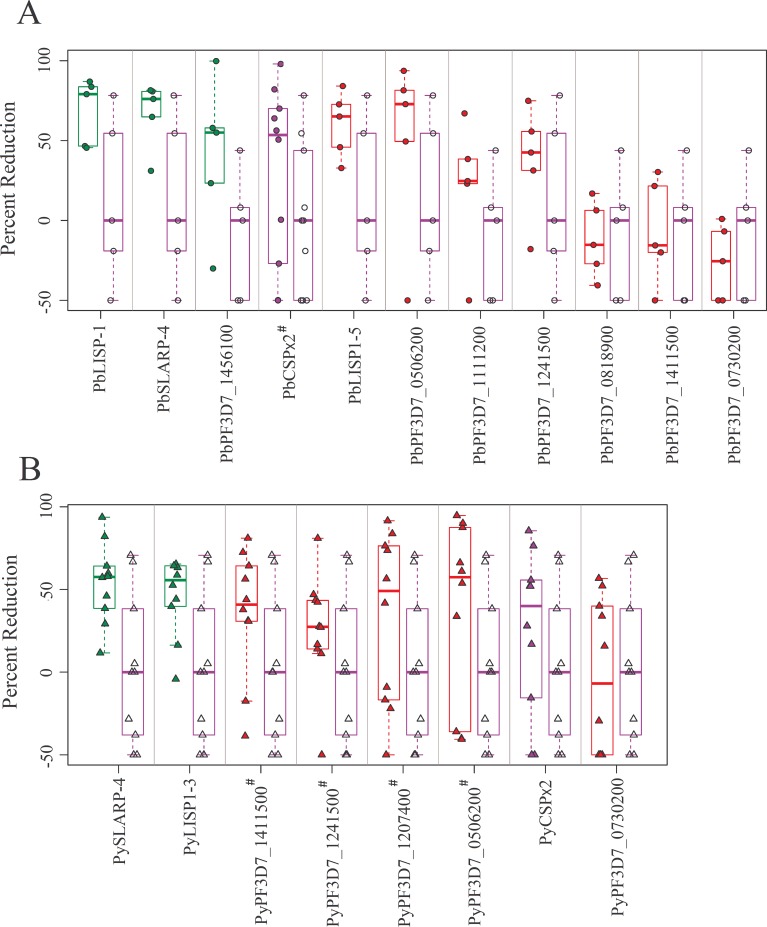
Novel antigens combined with CSP provide greater protection than CSP alone. (A) Each C57Bl/6 mouse (circles) was immunized with a combination of *Py*CSP DNA (2.5 μg) with novel antigen DNA (2.5 μg) by GG, using the schedule described in Fig 2. (B) Each BALB/c mouse (triangles) was immunized with a combination of *Pb*CSP DNA (10 μg) with novel antigen DNA (10 μg) by EP, using the same schedule used for the GG immunizations. Data were compared to the negative control group immunized with a combination of CSP and EV tested in the same immunization study. Significant reduction in LS parasite burden was determined by Kruskal-Wallis test followed by Mann-Whitney test and p<0.05 was considered as significant. Green box indicates p<0.05 and red box indicates p>0.05. A complete statistical analysis is provided in S6 Table.

Several antigens that demonstrated partial protection as individual immunogens in one or both of our animal models (7 *Py* and 9 *Pb* antigens) were selected for combination vaccine studies. Among these, *Py*SLARP-4 and *Py*LISP1-3 were protective by IM delivery, but protection by GG could not be interpreted due to QC criteria. Animals that received the combination of CSP and a novel antigen were compared to those immunized with CSP plus EV tested in the same immunization studies. None of the selected antigens interfered with protection induced by CSP alone, and in general, LS burden trended lower in mice that received combination vaccines. Two *Py* antigens (*Py*LISP1 and *Py*SLARP) and 3 *Pb* antigens (*Pb*LISP1, *Pb*SLARP and *Pb*PF3D7_1456100) conferred significantly greater protection than that seen with CSP plus EV (Fig 3 and S6 Table).

### Expression and localization of protective antigens

**Fig 4:**
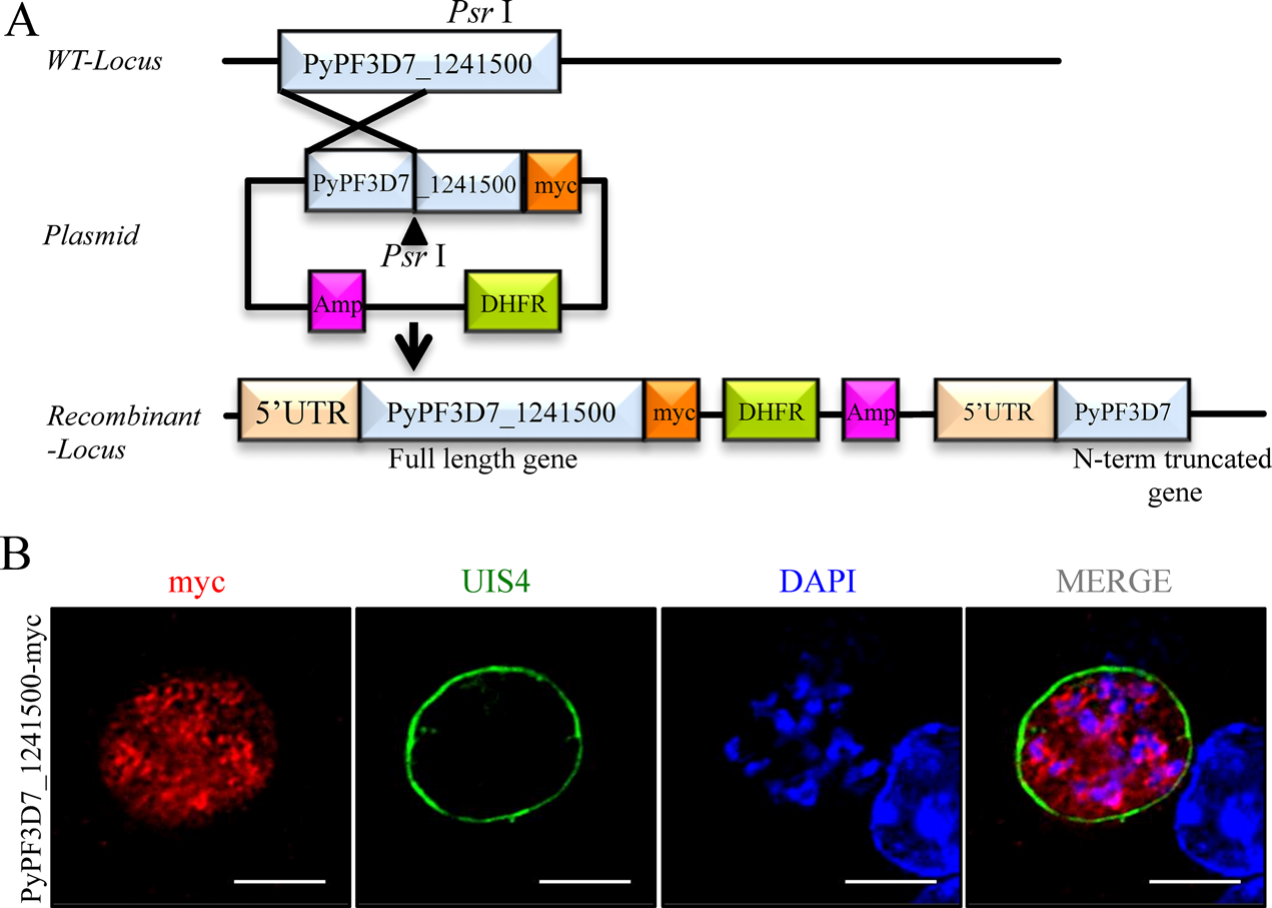
Expression of novel antigens by *Py* LS parasites. (A) Strategy for generation of myc-tagged *Py*PF3D7_1241500. (B) Immunofluorescence assay using *Py*17XNL grown 24h in HepG2-CD81 cells, showing expression of *Py*PF3D7_1241500 protein detected by Alexa-594 conjugated anti-myc antibody (red). UIS4 (green) was used as a PVM marker and DAPI to identify nuclei. Scale bar represents 10 μm.

We hypothesized that proteins that were secreted into the host cell cytosol or localized on parasitophorous vacuole membrane (PVM) might be over-represented among our protective antigens, since these proteins should be more accessible for processing and presentation to T cells. Based on *in silico* analyses, we did not find that antigens protective in both *Py* and *Pb* models were more likely to have putative signal peptides, transmembrane domains, or export motifs (PEXEL/VTS) (Table 2). Two proteins (LISP1 and SLARP) were previously reported to be expressed by LS parasites, and these respectively localize to PVM [26,27] and to early LS peri-nuclear region [24]. We studied protein expression of several of our novel vaccine candidates by myc epitope-tagging and immunofluorescence assays (Fig 4A). Myc-tagged *Py*PF3D7_1241500 was detected in 24h LS and localized to the parasite peri-nuclear area but was not exported (Fig 4B). *Py*PF3D7_0730200 also appeared in 24h LS and localized in parasite cytoplasm (S4A Fig), while *Py*PF3D7_0506200 localized to the parasite nucleus (S4B Fig). In summary, the novel candidate vaccine antigens are expressed as LS proteins, but most do not localize to PVM or host cell cytosol.

Additionally, we examined transcriptional profiles of genes in *Pf* for an association with protection in the mouse models. Among the antigens shown to be protective in both *Pb* and *Py* models as single immunogens, 5/6 were transcribed at higher levels in LS versus both SS and BS; only 8 of the other 15 antigens were similarly upregulated versus both SS and BS (Table 2). When analyzing the trajectory of transcription over the first 72 hours of *Pf* LS development in HC-04 cells, the group of antigens that conferred partial protection in both models showed an average increase (linear regression slope = 0.41/hr, p = 0.051) in their transcription, while antigens that failed to protect in one or both models showed a decline in their transcription (linear regression slope = -0.07/hr, p = 0.54). The linear regression trend, in terms of gene transcription, between the two groups is significantly different (p = 0.04) (S5 Fig).

## Discussion

Candidate pre-erythrocytic vaccine antigens have previously been selected by screening with sera or T cells from protected or malaria-exposed individuals, but these have demonstrated only modest or no protective efficacy in human clinical trials [48–52]. We have used a transcriptomic approach to identify novel pre-erythrocytic malaria antigens. Although the model of axenically cultured parasites does not fully mimic the development of intrahepatocytic parasites, the transcriptomes of these parasites appeared similar: 124/131 genes upregulated in axenic culture were also upregulated in parasitized HC-04 hepatic cells. We selected 21 genes that are transcribed at higher levels during LS compared to SS and/or BS parasites, and found that several conferred functional immunity that reduced LS burden in both the *Py* model used for initial screening as well as the *Pb* model used for confirmation. Two antigens conferred greater protection when used in combination with CSP versus CSP alone. The antigens that conferred protection in both models are expressed as proteins during *Pf* LS development and most are transcribed at higher levels in LS versus both SS and BS parasites (Table 2 and S5 Fig). These characteristics provide a rational basis in the future to identify additional candidate antigens for pre-erythrocytic vaccines, while this first generation of novel candidate antigens moves forward for further assessment as vaccines against human malaria.

As a group, the antigens that conferred protection in both murine models were encoded by genes that increase in transcription as early LS development proceeds. Interestingly, the genes that protected only in a single model, did not have this feature, and on average appeared to decrease in transcription (S5 Fig). This feature also distinguishes our protective antigens from CSP, which rapidly decreases in transcription after sporozoite invasion of hepatocytes [53]. The combination of CSP with antigens that are increasing in expression during LS development may enhance immune responses against a wider window of epitopes, which could explain why these combinations were superior to CSP alone.

We used DNA vaccination of mice to down select pre-erythrocytic genes that merit further investigation as candidate human vaccine antigens [45,54]. Mouse malaria models have been used extensively for assessment of candidate malaria vaccines. DNA vaccination of BALB/c mice with the *Pb* and *Py* orthologues of CSP confers partial protection against pre-erythrocytic malaria [45,47], providing a benchmark for the level of efficacy seen with new antigens. On average, CSP conferred the highest level of protection in both the *Py*–BALB/c model and the *Pb*–B6 model, consistent with evidence that CSP is the immunodominant antigen in response to whole sporozoite vaccines [18]. However, vaccination with several of our novel antigens also significantly reduced the LS parasite burden after sporozoite challenge, and in some cases their efficacy was similar to that observed with CSP (S5 Table). Ideally, these antigens could be developed and tested as individual immunogens in humans, and compared directly to CSP-based vaccines to assess their relative efficacy against experimental infection of humans.

Our primary goal in this work has been to identify antigens that could be combined with CSP in a more efficacious vaccine. CSP-based DNA vaccines are partially protective in these mouse models, and thus well-suited for this assessment. In general, LS parasite burden was lower in animals that received the combination of our novel antigens with CSP, compared to CSP combined with EV. Additional studies are warranted with all the antigens to confirm the significance of these differences. Immunization with LISP1 [26,27] or SLARP [24,25] in combination with CSP afforded greater protection than CSP alone in both animal models, lending strong support for their further development in a combination vaccine. Notably, *Pb*SLARP and *Py*LISP1 antigens did not perform well when used alone for immunizations. In earlier studies, pre-erythrocytic antigens that conferred a modest degree of protection alone nevertheless achieved a relatively high degree of protection as a combination vaccine in mice [55], similar to our results here.

Two of our candidates were previously shown to be expressed by intrahepatic parasites: SLARP localizes to the cell interior of LS parasites [24], while LISP1 appears to be associated with the PVM surrounding the developing LS [26,27]. Among the antigens that we examined by myc-tagging, all were confirmed to be expressed as protein by *Py* LS parasites, but their localization varied. *Py*PF3D7_1241500 localized to the peri-nuclear area, *Py*PF3D7_0730200 appeared in parasite cytoplasm (S4A Fig), and *Py*PF3D7_0506200 localized to the parasite nucleus (S4B Fig). Thus, expression but not localization during LS development is a shared feature of our protective antigens. Although we speculated that protein export might enhance processing and presentation to T cells, we could not conclude that antigens with relevant features, including PEXEL motifs, transmembrane domains and signal sequences, were more likely to confer protection in our limited dataset. Five (PF3D7_1411500, PF3D7_0730200, PF3D7_1241500, PF3D7_1207400, and LISP1) out of 6 antigens that conferred protection in both models lacked any of these predicted features (Table 2).

Little is known about the function of most antigens that demonstrated protective activity in these studies. The nuclear protein SLARP is a transcriptional regulator that controls expression of several known LS genes, such as UIS3, UIS4, p36, and EXP-1 [25,56]. Products of UIS genes often function as components of PVM. Targeted deletion of SLARP leads to fast terminal degradation of many UIS and additional non-UIS transcripts [25,56]. SLARP^-^ mutants are able to traverse hepatocytes, invade host cells and form PVM, but are unable to initiate early LS development. The critical role of SLARP in early LS development is an attractive feature for a vaccine candidate.

LISP1 knockout parasites develop normally through blood and mosquito stages, but demonstrate a severe defect in egress from hepatocytes [26], which raises the possibility that parasite egress may be a novel target for pre-erythrocytic vaccines. We recently showed that the BS schizont protein SEA-1 has a role in parasite egress from erythrocytes, and antibodies to SEA-1 reduce parasite burden and severe malaria risk in mouse models and in human immunoepidemiology studies [57]. Antibodies induced by RTS,S vaccine have been associated with protection from infection in humans [10,11], and the strongest correlate of protection induced by RAS vaccine is antibody activity that inhibits sporozoite invasion of hepatocytes [12]. Therefore novel pre-erythrocytic antigens that confer protection through antibody, at least in part, might be appealing partners to combine with RTS,S.

Evidence supporting the effector function of CD8^+^ T cells against pre-erythrocytic stage of infection is based on RAS vaccination studies in animals [15,58,59] as well as humans [60]. Therefore, we examined the impact of CD8 depletion on levels of protection after vaccination. We used CB6F1 mice as an additional malaria model, because their genetic diversity relative to their parental strains might permit greater diversity in immune responsiveness. Among four of our LS antigens that conferred significant protection in pilot studies with this model, we found that while some antigens (*Py*PF3D7_1456100 and *Py*SLARP) induced CD8^+^ T cells required for protection, others (*Py*PF3D7_1207400) relied only partially on CD8^+^ T cells, and in one case (*Py*PF3D7_0506200), CD8^+^ T cells did not contribute to protection. The results indicate that the antigens identified by transcriptome profiling of LS parasites can mediate protection through different immune mechanisms that do not always involve CD8^+^ T cells.

In summary, we used transcriptome profiling and DNA vaccine immunization to identify 6 novel antigens that show efficacy in two murine malaria models by inducing diverse immune responses that reduce LS parasite burden. Two antigens in combination with CSP showed significantly greater efficacy than CSP alone in both models. In general, these antigens had the common features of LS expression, and of increasing transcription during early LS development. The antigens LISP1 and SLARP should be given priority for evaluation in combination with a CSP-based vaccine like RTS,S to prevent malaria infection in humans.

## Supporting Information

S1 Fig EP/IM DNA immunization and reduction of LS parasite burden post-sporozoite challenge. (**A**) Meta-analyses of 5 independent immunization experiments and resulting LS parasite burden reduction in *Py* model with IM immunization. (**B**) Meta-analyses of 7 independent immunization experiments and resulting LS parasite burden reduction in *Py* model induced by EP immunizations. (**C**) Meta-analyses of 4 independent immunization experiments and resulting LS parasite burden reduction in *Pb* model induced by EP immunizations. Each square or triangle represents one BALB/c or B6 mouse, respectively. Green color indicates significant difference as compared to EV immunized groups tested in the same immunization studies (p<0.05). Red color indicates p>0.05 and therefore no significant difference in LS burden as compared to EV immunized mice. CSP (purple) was used as positive control. EV (black) was used as negative control.
(PDF)

S2 Fig Novel LS antigens confer protection by different mechanisms. (A) Three of four novel antigens delivered as DNA vaccines induced IFN-γ responses recalled in CD8^+^ T cells by peptides predicted to be CD8^+^ T cell epitopes for either H-2^b^ or H-2^d^ mouse haplotypes. Spleens from immunized CB6F1 mice (5/group) were harvested 2 weeks after the final immunization and re-stimulated *in vitro* with peptides. Shown are the percentages of IFN-γ CD8^+^ T cells. Data points represent individual mice. (B) Protection induced by some, but not all, *Py* LS DNA antigens is mediated by CD8^+^ T cells. Control (black bars) and experimental (gray bars) CB6F1 mice were immunized 3 times at 3 week intervals with *Py* DNA delivered by GG. 2 weeks after the last boost mice were challenged with 10,000 *Py* sporozoites intravenously and the livers were harvested 40h after the challenge. Experimental group mice were treated with anti-CD8β depleting antibody 26-28h before the challenge. Protection was defined as a significant reduction of parasite burden in the livers compared to mice immunized with EV, * P<0.05
(PDF)

S3 Fig Depletion of CD8^+^ T cells. CD8^+^ T cells were specifically depleted by intraperitoneal injection of 100μg Rat anti-mouse-CD8β antibody approximately 26-28hrs prior to challenge. (A) Representative flow plots demonstrating depletion of CD8^+^ T cells from PBMCs 24hrs post depletion. (B) Percent depletion of CD8^+^ T cells calculated compared to non-depleted control mice from the same group. Data from individual mice is shown. EV and *Py*CSP represent data compiled from several experiments.
(PDF)

S4 Fig Determination of LS expression of two novel PEVA in *Py* by myc-tagging and IFA with 24h LS parasite. Myc-tagged transgenic parasites were generated and designated as *Py*PF3D7_0730200-myc and *Py*PF3D7_0506200-myc. (A) Immunofluorescence of *Py*PF3D7_0730200-myc expressed by 24h LS *Py*17XNL grown *in vitro*. Detection of *Py*PF3D7_0730200-myc gene expression by Alexa-488 conjugated anti-myc antibody (green) confirms LS expression of *Py*PF3D7_0730200. UIS4 (red) was used as a PVM marker. (B) Immunofluorescence of *Py*PF3D7_0506200 expressed by 24h LS *Py*17XNL grown *in vitro*. Detection of *Py*PF3D7_0506200 gene expression by Alexa-594 conjugated anti-myc antibody (Red) confirms LS expression of *Py*PF3D7_0506200. UIS4 (green) used as a PVM marker. DAPI was used in both cases to identify nucleus. Scale bar represents 10 μm.
(PDF)

S5 Fig Transcription profile (quantified as expression rank percentile) of *Pf* LS genes that protected in both mouse malaria models compared to genes that failed to protect in one or both models. qPCR performed on RNA isolated from different parasite stages, including SS, LS at 24h, 48h, and 72h post infection, and mixed BS, to quantify the expression of each selected LS gene, normalized to expression of the parasite GAPDH gene. Graph represents box-plot of the meta-analysis of the expression rank percentile of 6 genes that protected in both parasite models (right panel) and 15 genes that failed to protect in one (n = 14) or both (n = 1) models (left panel).
(PDF)

S1 Table Primer used in qPCR used to confirm and quantify transcription of 131 candidate genes. List of primer used to verify the expression profile of selected candidate genes in SS, BS, and 24h, 48h and 72h of LS.
(PDF)

S2 Table Primers to amplify inserts for DNA vaccine constructs. Table shows the list of primer used for generation of vaccine construct. Start codon in forward primer and stop codon in reverse primer are shown in lower case letter.
(PDF)

S3 Table Peptides used for in vitro stimulation of splenocytes from DNA immunized mice. Table shows the list of predicted peptides from indicated antigens.
(PDF)

S4 Table Primers to amplify construct inserts for myc-epitope tagging of proteins in transgenic *Py* parasite. Table shows the primer sequence used to generate recombination construct for myc-tagging of selected *Py* protein. Gene ID of selected *Py* protein is shown in the primer name.
(PDF)

S5 Table LS Burden reduction after vaccination with individual antigens. LS burden in mice vaccinated with the indicated antigen was compared to that in mice vaccinated with EV. Antigens are listed from greatest to smallest reduction in LS burden. Only antigens that significantly reduced LS burden are shown. CSP is shown in bold.
(PDF)

S6 Table LS burden reduction with CSP combination vaccines. LS burden in mice vaccinated with a combination of CSP and the indicated antigen, was compared to that in mice vaccinated with CSP in combination with EV. Antigens are listed from greatest to smallest reduction in LS burden. Only antigens that significantly reduced LS parasite burden are shown. Doubling the CSP dose did not significantly reduce LS parasite burden beyond that achieved with CSP in combination with EV.
(PDF)
