# Correction: Identification of Novel Pre-Erythrocytic Malaria Antigen Candidates for Combination Vaccines with Circumsporozoite Protein

**DOI:** 10.1371/journal.pone.0165489

**Published:** 2016-10-20

**Authors:** C Speake, A Pichugin, T Sahu, V Malkov, R Morrison, Y Pei, L Juompan, N Milman, S Zarling, C Anderson, NJ MacDonald, S Wong-Madden, J Wendler, A Ishizuka, ZW MacMillen, V Garcia, SH Kappe, U Krzych, PE Duffy

Dr. Nicholas J. MacDonald should be included in the author byline. Dr. MacDonald should be listed as the eleventh author, and with the affiliation 3: Laboratory of Malaria Immunology and Vaccinology, National Institute of Allergy and Infectious Diseases, National Institutes of Health, Bethesda, Maryland, United States of America. The contributions of this author are as follows: Conception and design of the work, acquisition of data, or analysis and interpretation of data; drafting the article or revising it critically for important intellectual content; final approval of the version to be published; agreement to be accountable for all aspects of work; conceptualization; methodology; investigation; and resources.
