# Supplementary figures and images for: Identification of Novel Pre-Erythrocytic Malaria Antigen Candidates for Combination Vaccines with Circumsporozoite Protein
Source: PLoS One. 2016 Jul 19;11(7):e0159449. doi: 10.1371/journal.pone.0159449 (PMC4951032; doi:10.1371/journal.pone.0159449)

A

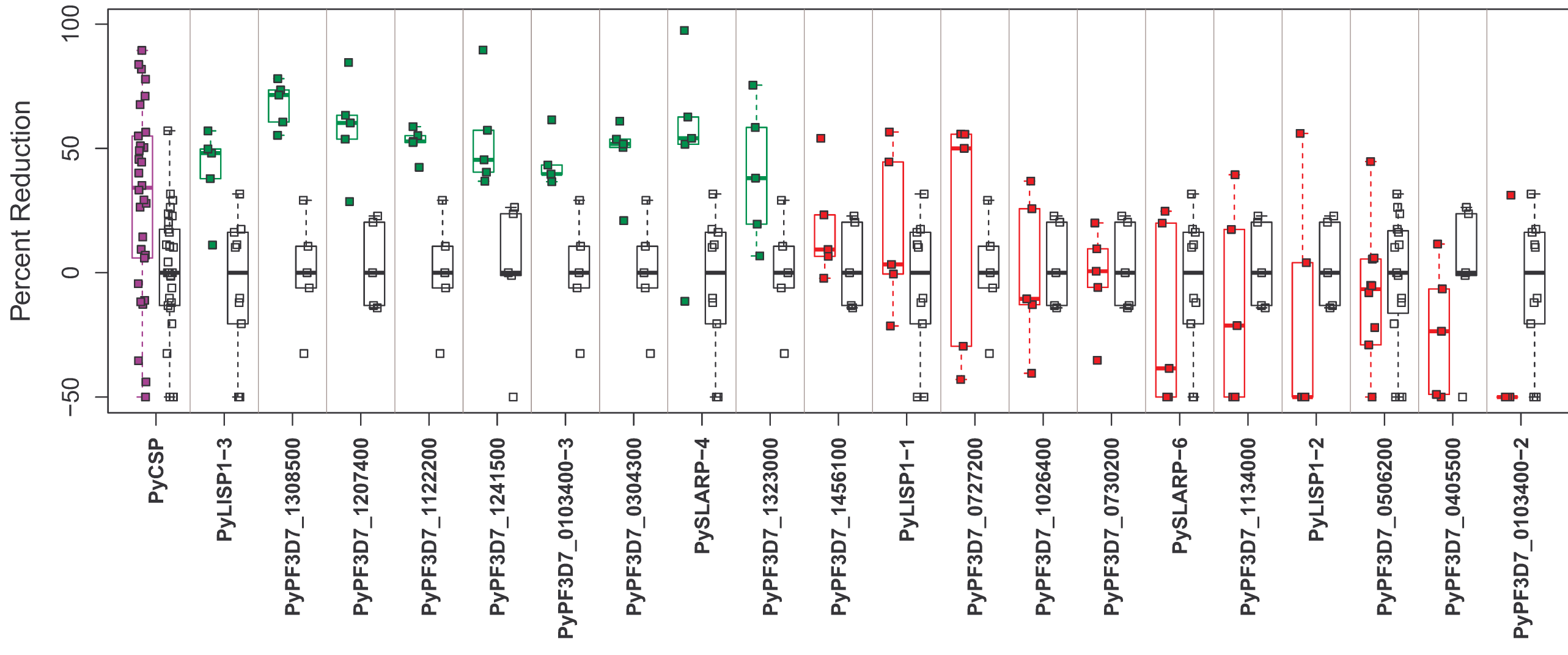

B

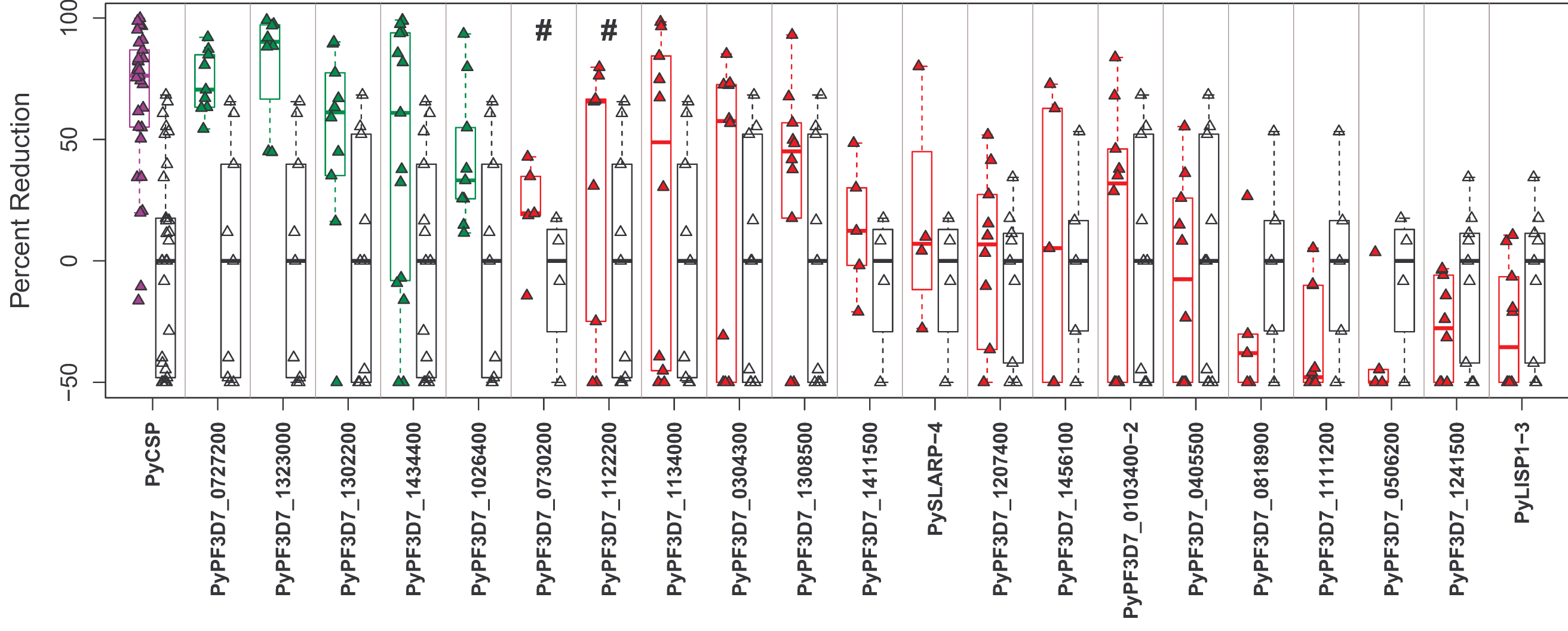

C

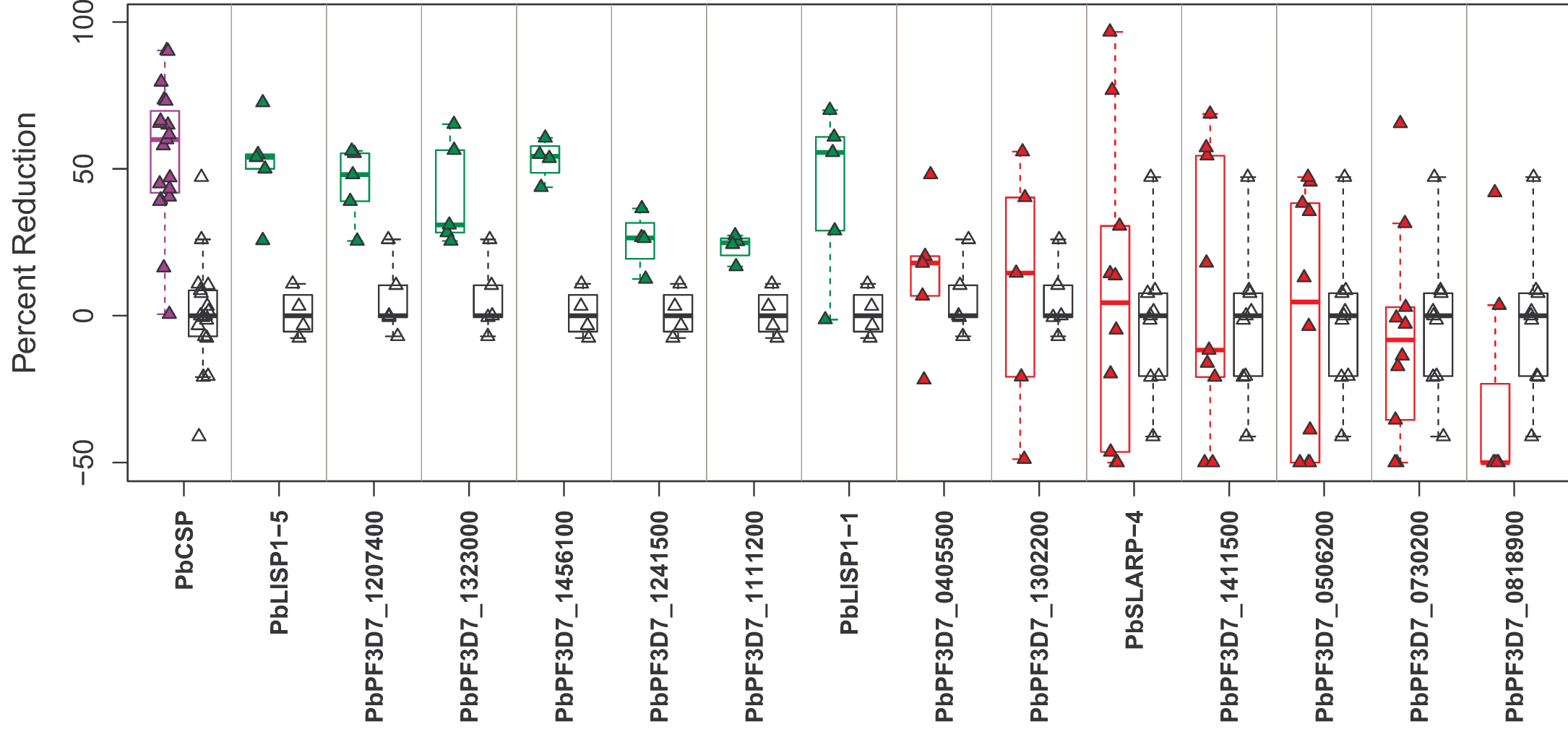

Supplement: S1 Fig — (A) Meta-analyses of 5 independent immunization experiments and resulting LS parasite burden reduction in Py model with IM immunization. (B) Meta-analyses of 7 independent immunization experiments and resulting LS parasite burden reduction in Py model induced by EP immunizations. (C) Meta-analyses of 4 independent immunization experiments and resulting LS parasite burden reduction in Pb model induced by EP immunizations. Each square or triangle represents one BALB/c or B6 mouse, respectively. Green color indicates significant difference as compared to EV immunized groups tested in the same immunization studies (p<0.05). Red color indicates p>0.05 and therefore no significant difference in LS burden as compared to EV immunized mice. CSP (purple) was used as positive control. EV (black) was used as negative control. (PDF) [file pone.0159449.s001.pdf]

A

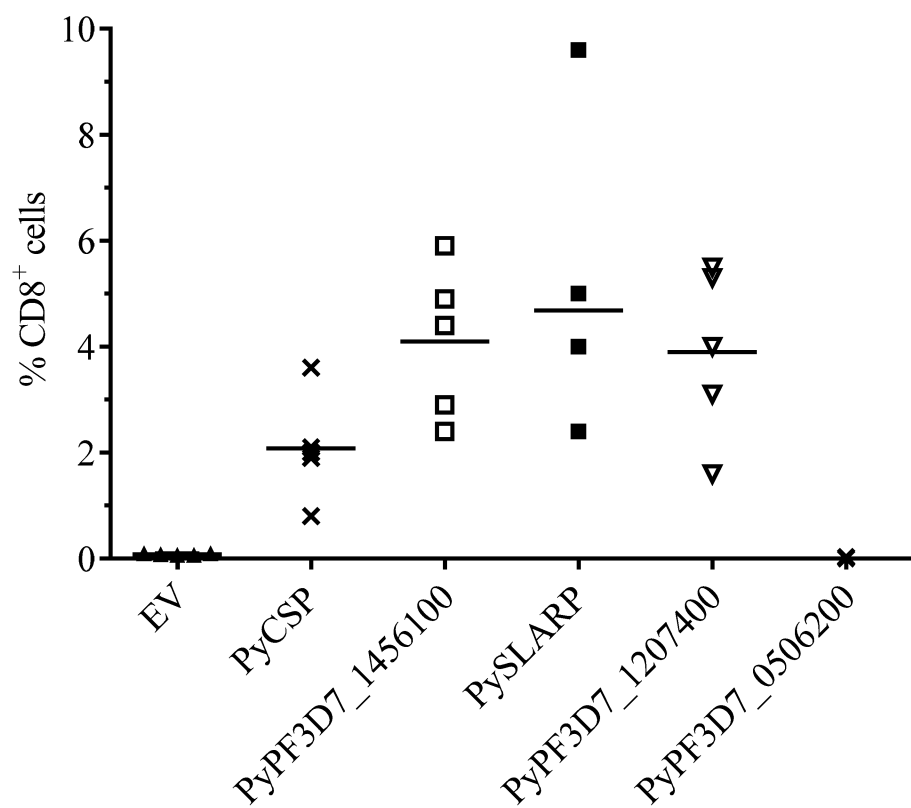

B

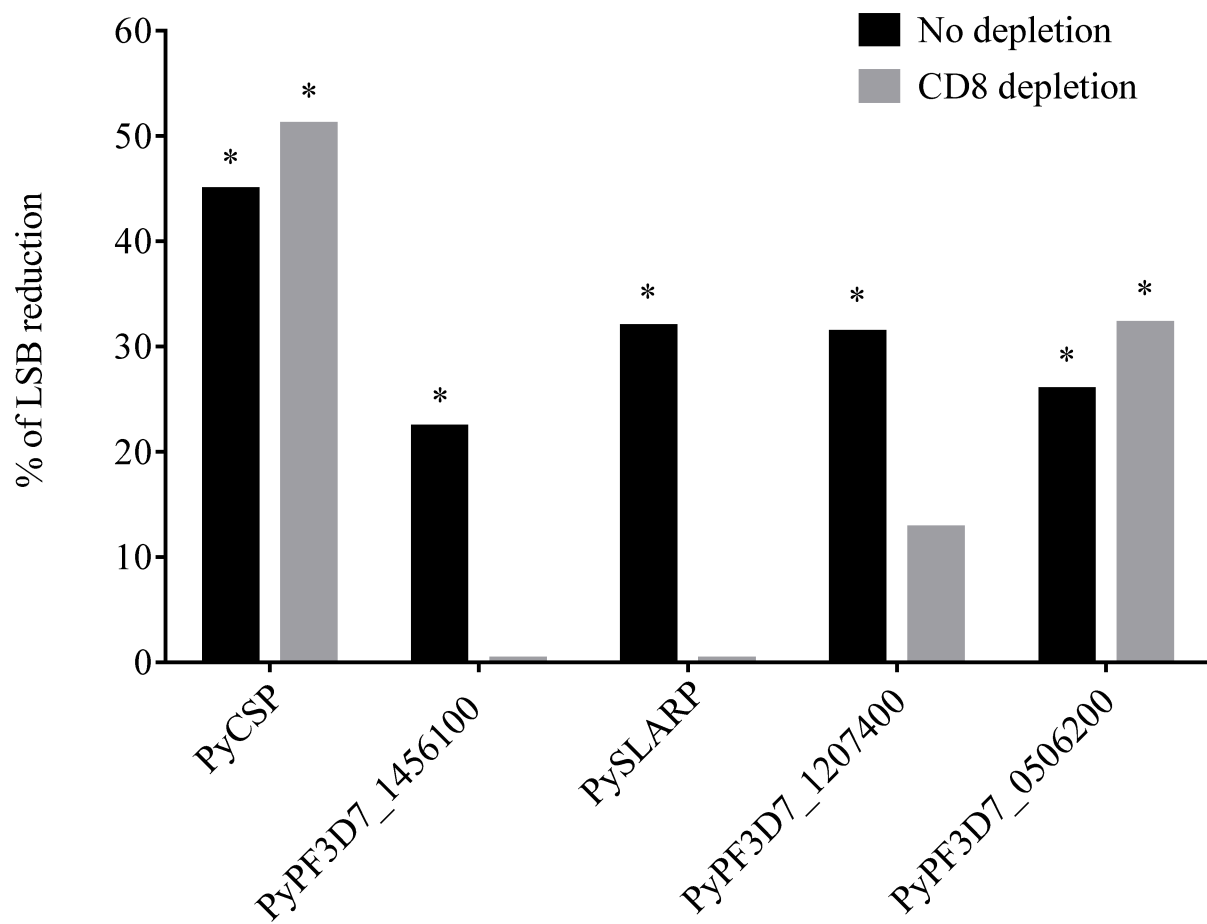

Supplement: S2 Fig — (A) Three of four novel antigens delivered as DNA vaccines induced IFN-γ responses recalled in CD8+ T cells by peptides predicted to be CD8+ T cell epitopes for either H-2b or H-2d mouse haplotypes. Spleens from immunized CB6F1 mice (5/group) were harvested 2 weeks after the final immunization and re-stimulated in vitro with peptides. Shown are the percentages of IFN-γ CD8+ T cells. Data points represent individual mice. (B) Protection induced by some, but not all, Py LS DNA antigens is mediated by CD8+ T cells. Control (black bars) and experimental (gray bars) CB6F1 mice were immunized 3 times at 3 week intervals with Py DNA delivered by GG. 2 weeks after the last boost mice were challenged with 10,000 Py sporozoites intravenously and the livers were harvested 40h after the challenge. Experimental group mice were treated with anti-CD8β depleting antibody 26-28h before the challenge. Protection was defined as a significant reduction of parasite burden in the livers compared to mice immunized with EV, * P<0.05 (PDF) [file pone.0159449.s002.pdf]

**control**

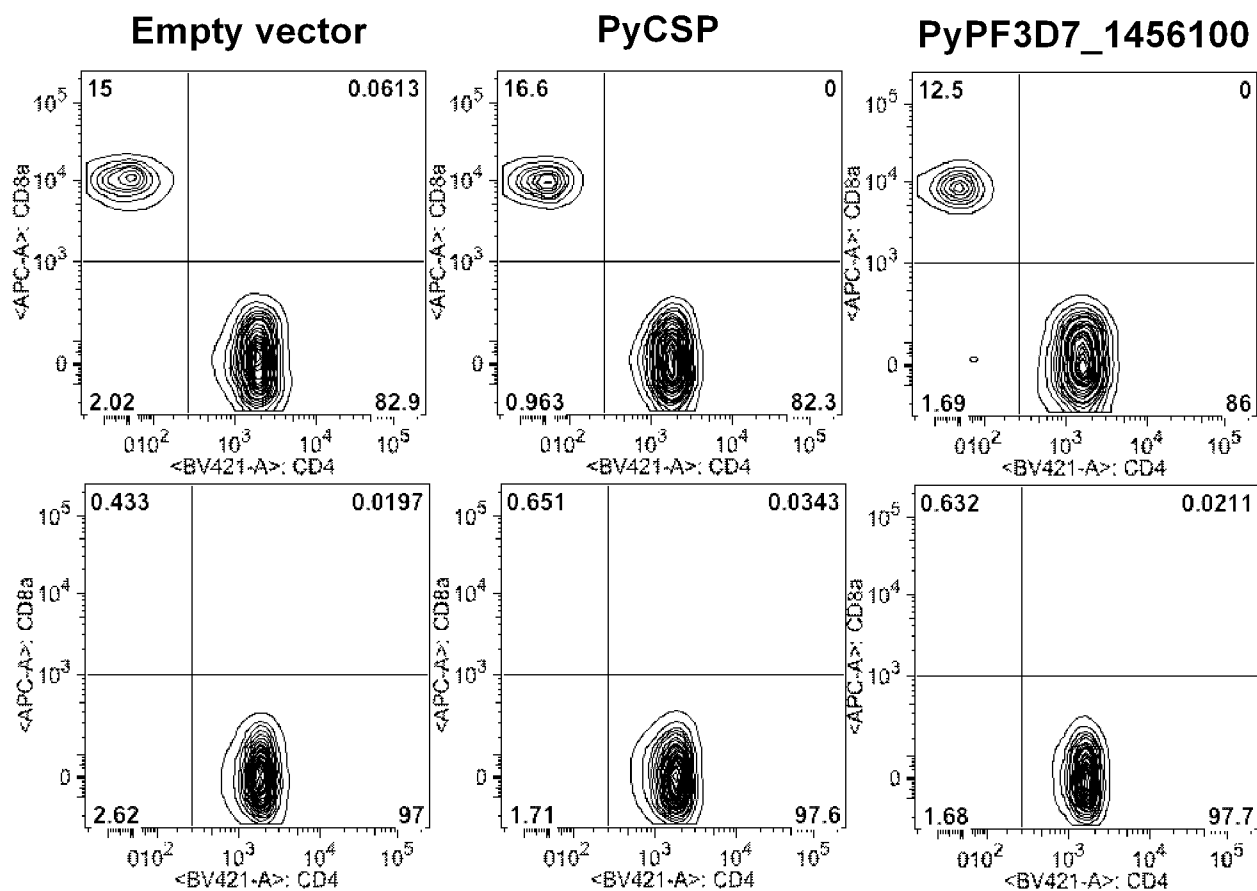

**% depletion**

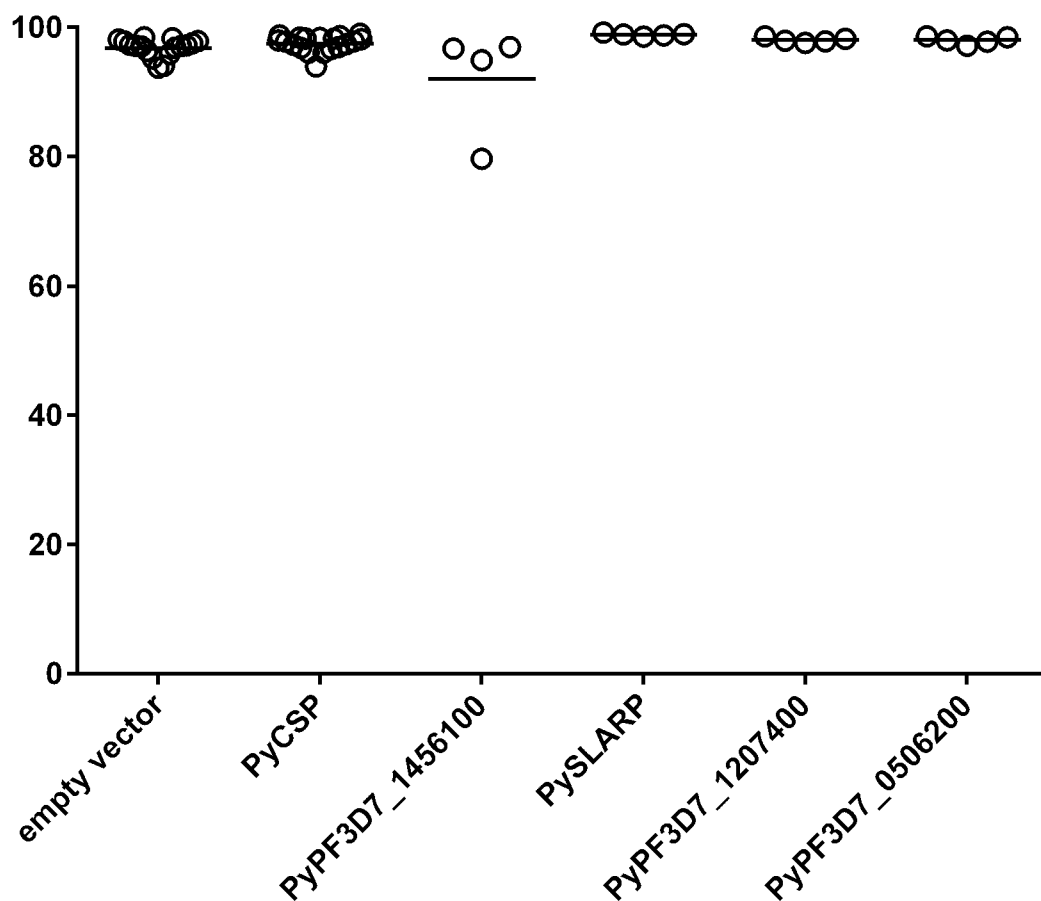

Supplement: S3 Fig — CD8+ T cells were specifically depleted by intraperitoneal injection of 100μg Rat anti-mouse-CD8β antibody approximately 26-28hrs prior to challenge. (A) Representative flow plots demonstrating depletion of CD8+ T cells from PBMCs 24hrs post depletion. (B) Percent depletion of CD8+ T cells calculated compared to non-depleted control mice from the same group. Data from individual mice is shown. EV and PyCSP represent data compiled from several experiments. (PDF) [file pone.0159449.s003.pdf]

A

PyPF3D7\_0730200-myc

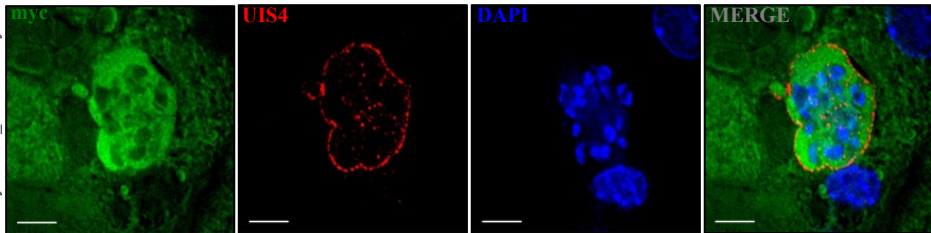

B

PyPF3D7\_0506200-myc

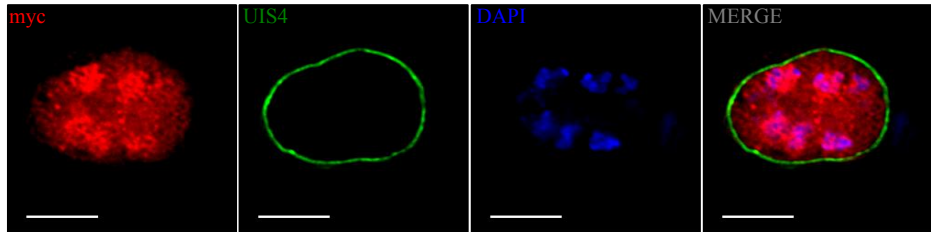

Supplement: S4 Fig — Myc-tagged transgenic parasites were generated and designated as PyPF3D7_0730200-myc and PyPF3D7_0506200-myc. (A) Immunofluorescence of PyPF3D7_0730200-myc expressed by 24h LS Py17XNL grown in vitro. Detection of PyPF3D7_0730200-myc gene expression by Alexa-488 conjugated anti-myc antibody (green) confirms LS expression of PyPF3D7_0730200. UIS4 (red) was used as a PVM marker. (B) Immunofluorescence of PyPF3D7_0506200 expressed by 24h LS Py17XNL grown in vitro. Detection of PyPF3D7_0506200 gene expression by Alexa-594 conjugated anti-myc antibody (Red) confirms LS expression of PyPF3D7_0506200. UIS4 (green) used as a PVM marker. DAPI was used in both cases to identify nucleus. Scale bar represents 10 μm. (PDF) [file pone.0159449.s004.pdf]

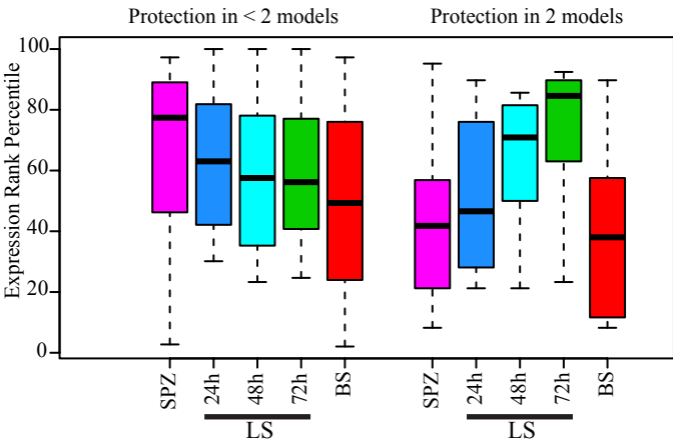

Supplement: S5 Fig — qPCR performed on RNA isolated from different parasite stages, including SS, LS at 24h, 48h, and 72h post infection, and mixed BS, to quantify the expression of each selected LS gene, normalized to expression of the parasite GAPDH gene. Graph represents box-plot of the meta-analysis of the expression rank percentile of 6 genes that protected in both parasite models (right panel) and 15 genes that failed to protect in one (n = 14) or both (n = 1) models (left panel). (PDF) [file pone.0159449.s005.pdf]
