# Supplementary material for: Identification of Novel Pre-Erythrocytic Malaria Antigen Candidates for Combination Vaccines with Circumsporozoite Protein
Source: PLoS One. 2016 Jul 19;11(7):e0159449. doi: 10.1371/journal.pone.0159449 (PMC4951032; doi:10.1371/journal.pone.0159449)
Supplement: S1 Table — List of primer used to verify the expression profile of selected candidate genes in SS, BS, and 24h, 48h and 72h of LS. (PDF) [file pone.0159449.s006.pdf]

**S1 Table. Primer used in qPCR used to confirm and quantify transcription of 131 candidate genes**

| Primer Name | Primer Sequence |
|---|---|
| PF3D7_1308500-1F | TTTAATACAATGTGATGTTTTATATCC |
| PF3D7_1308500-1R | CACCTTCTATAATAAGAGATTGTTTG |
| PF3D7_1308500-2F | TTAATGTGACTAAGAGGGATGAG |
| PF3D7_1308500-2R | GTATTTCTTTGCATCTTTTCAAC |
| PfLISP1-1F | GATATCACCAACGATATTTCACA |
| PfLISP1-1R | TAACTGGAGGATATGGGCTTA |
| PfLISP1-2F | ATTTGTCTATTGCCGATGTG |
| PfLISP1-2R | ATTATGTGAAATATCGTTGGTGA |
| PfCSP-1F | AGAACCAAGTGATAAGCACATAA |
| PfCSP-1R | ACTTGAATACCATTTCCACAAG |
| PfCSP-2F | AACCGAAATGTAGATGAAAATG |
| PfCSP-2R | GGACCATTCAGTTGAAAGAGA |
| PfHSP70-A-1F | AATTGAAAGAAACACAACCATAC |
| PfHSP70-A-1R | TCACCTTCATATACTTGAATTAAGAC |
| PfHSP70-A-2F | ACTTATGCTGATAACCAACCAG |
| PfHSP70-A-2R | TTCGATTTGTGGTACCTTTC |
| PF3D7_1026400-1F | CACCTTACAGAATTACCTTTTCC |
| PF3D7_1026400-1R | TTCGTAATCTTGTCTTCATTTTC |
| PF3D7_1026400-2F | CATAATACATTAACAACTGGTGGTC |
| PF3D7_1026400-2R | TAAATATGTACCATCTGCATTCC |
| PF3D7_1111200-1F | AACAAATAACTGGGTAAACACAC |
| PF3D7_1111200-1R | GAGCATCTTCATATTTATCTTCAAC |
| PF3D7_1111200-2F | TGTATTAACAACACACTTGATGAAA |
| PF3D7_1111200-2R | CTTCCTTTATTTTATATTCTCTCCATT |
| PF3D7_1122200-1F | GAACATATGAAAGAAAAAATAGAGAATGTAAAT |
| PF3D7_1122200-1R | CTTGGATCCTTATACATCCATACATAACTTACCATC |
| PF3D7_0304300-1F | GAACCATGGTAAAATTTTCAAAACATGTCAG |
| PF3D7_0304300-1R | CTTGGATCCTTTATTTTTTCAAATAATGCTTTAAATC |
| PF3D7_1241500-1F | AACATGAACTTAAATACGGAAAATA |
| PF3D7_1241500-1R | TTACTTCGAAAACATTATGAAACA |
| PF3D7_0518700-1F | CACCCAAAACGATAATAAATGT |
| PF3D7_0518700-1R | ATTTGATTTGCTCCTAATATGC |
| PF3D7_0518700-2F | GAGAAAGAGCATATTAGGAGCA |
| PF3D7_0518700-2R | TTATTGGAATTTTCATTGTTCTG |
| PfSLARP-1F | AGCCAGGATGGAACTTGTA |
| PfSLARP -1R | TGTTATATGTTTCAAAGGATGGA |
| PfSLARP -2F | ACGAACAAAGAGTACATTAACATTT |
| PfSLARP -2R | AGTTCCATCCTGGCTTTTATT |
| PF3D7_0103400-1F | AAAAGAGAAGGAGCATATTTCG |
| PF3D7_0103400-1R | TATTTCCTGTCTTGAAAGCATC |
| PF3D7_0103400-2F | TTTATTCATGACGGGTGTTAG |
| PF3D7_0103400-2R | AATATACCACACGTTTCTGTCG |
| PF3D7_1411500-1F | AACACAATCCATAAATAGTGTGAA |

| | |
|---|---|
| PF3D7_1411500-1R | CATGTTCTTTTGGGCTTATTT |
| PF3D7_1411500-2F | GATGGTGAAGATAATCTAAGTCAAG |
| PF3D7_1411500-2R | CTTGTAATTTATTGTAAGGCTGTTC |
| PF3D7_1434400-1F | GATTACACAAATCATATCCCAAC |
| PF3D7_1434400-1R | TTTTGGCTAGCATTATCTTTACC |
| PF3D7_1434400-2F | AAATTTCGAAGTAGGGAATTATG |
| PF3D7_1434400-2R | TGTAAAGTGTCACATGAGCAAG |
| PF3D7_1302200-1F | CTTGTTTGCGCACATATTTT |
| PF3D7_1302200-1R | TTGTTGTGTTCTTCTATTGCTTT |
| PF3D7_1302200-2F | ATTGATAAAGCAATAGAAGAACACA |
| PF3D7_1302200-2R | TATAAATCATCTTCTCGCGATT |
| PF3D7_1302200-3F | ATATTGGGATTAGGATATTATGGA |
| PF3D7_1302200-3R | AGCACCATCTTGATATTTTATATTG |
| PF3D7_0730200-1F | AGTGACATAACAAAAGATTCCAC |
| PF3D7_0730200-1R | TCATGTATTGCGTGCTTATG |
| PF3D7_0730200-2F | TTTTAATAATCAACGAATTGAGTG |
| PF3D7_0730200-2R | ATAATTCTACTACCCTTGAAATGC |
| PF3D7_1444800-1F | GTATTATAAAGCTGGTGCAAGG |
| PF3D7_1444800-1R | AGATGCATATCTAGCCAATCC |
| PF3D7_1444800-2F | ATTTATCAATTCACGAAACTGC |
| PF3D7_1444800-2R | TTTGAGTTACAACTGCACAAAC |
| PF3D7_1444800-4F | CCAGGTATTAAGGTTGATAAAGG |
| PF3D7_1444800-4R | AATACTCTTTGCATCTTTCTGC |
| PF3D7_0624000-1F | AATGTTTTACAAAGTGCATGTTC |
| PF3D7_0624000-1R | GCCTTAGCAACTTTCCTACTATC |
| PF3D7_0624000-2F | TTAGTTATGGATTGGTATTCAGC |
| PF3D7_0624000-2R | TACGATTTCACCTAAGTAAGCAC |
| PF3D7_1203700-1F | ATTAAACAATTAAGCAAACATGAAG |
| PF3D7_1203700-1R | AAGATAATAGTCAACTGCATAGGG |
| PF3D7_1203700-2F | TAATAGAAGGAGACTATGAAGTTGC |
| PF3D7_1203700-2R | TTGCTTTCACTCTCGATAATAATAC |
| PF3D7_1203700-3F | GAGCCAGTGTTATTACATACAGAAG |
| PF3D7_1203700-3R | AAGAAACTATCCCTATTAACTGTTTG |
| PF3D7_0506200-1F | CGGTTTCTATAAGGAATGCTG |
| PF3D7_0506200-1R | TCTAGTACCTGTTAACATAATTCTTCC |
| PF3D7_0506200-2F | ACCATAATGATTACGATAACGAG |
| PF3D7_0506200-2R | TTTCATAATTTTGTTCTTCATAAGC |
| PF3D7_0405500-1F | GCTCTAAATGTTATGCTATAAAGGAC |
| PF3D7_0405500-1R | CAAGAATATTTCTCATTCGTACATC |
| PF3D7_0405500-2F | AGTAAGAAAAGGTTAAATGAAGCA |
| PF3D7_0405500-2R | TCACATCATACAAAAGAAATGG |
| PF3D7_0321700-1F | AACTTCAATCCTATAAGGAAATAAAAG |
| PF3D7_0321700-1R | TCTATTTGTATTTGAGCAATATGTTTA |
| PF3D7_0321700-2F | AAAAGTAGAATCCTTAGAACATGC |
| PF3D7_0321700-2R | TTCGAATTGGTTTTAATATCTTG |
| MSP1-p19-1F | GTGTTGAAAATCCAAATCCTAC |

| | |
|---|---|
| MSP1-p19-1R | TCGAAAAGTGGATAAGAATCAG |
| PF3D7_1401700-1F | AAAGAGATGAACAGAAAAGAAATG |
| PF3D7_1401700-1R | GATGAATTCTCGCTTTTATACG |
| PF3D7_1401700-2F | TTTGGTAGTTGATGTTCAAAATAG |
| PF3D7_1401700-2R | AAATTATTACCCATGTTATTTATTCC |
| PF3D7_0601700-1F | AAATACCCCACATGGTATAAATC |
| PF3D7_0601700-1R | TGATGCTTTGTCTTTAACTAAACC |
| PF3D7_0601700-2F | TGGTTTAGTTAAAGACAAAGCATC |
| PF3D7_0601700-2R | TTTCTGTAAACCTTTTCTTATGTTC |
| PF3D7_1456100-1F | CCAGACATTATATATTTTGACGAAT |
| PF3D7_1456100-1R | AATTGGCTTTGTTATTCATATTG |
| PF3D7_1456100-2F | ATGATAGATTCATTAAAGGACTAAAGA |
| PF3D7_1456100-2R | TGGGTAAAAGGAGAATTGATAAG |
| PF3D7_0917900-1F | AGCATTAACCAAAGATAATCACC |
| PF3D7_0917900-1R | TCAGCTTCAACATGTAAGATACC |
| PF3D7_0917900-2F | TTTAGGAAAGTTTGAATTATCTGG |
| PF3D7_0917900-2R | TTTACCTGTACCTTTGTCTTCAG |
| LSA1-PF3D7_1036400-4F | CAGAGGATTTATATGGTCGTTTAG |
| LSA1-PF3D7_1036400-4R | CTCTACTATTCCCTCTGTTGTCC |
| LSA1-PF3D7_1036400-5F | ATCTGCTGACATACAAAATCATAC |
| LSA1-PF3D7_1036400-5R | TCGTCATATTCAGCACTTATTTC |
| LSA3-PF3D7_0220000-1F | TAGGGGATATGGATAAGGAAAG |
| LSA3-PF3D7_0220000-1R | TTCCAATTTAGGTCTTTGAGC |
| LSA3-PF3D7_0220000-2F | ACTTGGAGAAAGAGTTGAATCC |
| LSA3-PF3D7_0220000-2R | TTTGGTTCTTCTTTAACCTCTTC |
| PF3D7_1140000-1F | GAGAATGTATCCTGGAAAGTAGC |
| PF3D7_1140000-1R | ATCTTCTACATTTTGTAAACTCCTG |
| PF3D7_1140000-2F | GGATCTTCTACATTACCCCTATG |
| PF3D7_1140000-2R | AAATGGTTTCAGTTGATACAGG |
| PF3D7_0915200-1F | TAGAGGGCGATTATTTATTGG |
| PF3D7_0915200-1R | AAAGTGGTAAATTTTCTGTATCATC |
| PF3D7_0915200-2F | CTTAAAAGAAATCGTGACCTCTC |
| PF3D7_0915200-2R | CTCCTTTATGTTATCTCTATCACATTC |
| PF3D7_0631500-1F | ATAATAATATGGGAAATGAGATGG |
| PF3D7_0631500-1R | TCTGATGGGTCTGTTAATGTATAG |
| PF3D7_0631500-2F | GTTTTATCAACCATGACATTAGC |
| PF3D7_0631500-2R | TTCTGTGTGTATATTTTGTCCTTC |
| PF3D7_0631500-3F | TATGAATGATAAATGGGAACATC |
| PF3D7_0631500-3R | AATTATTTTCTTCGCCATCTG |
| PF3D7_0631500-4F | AAAGACAATACAAAAGTGGATAATG |
| PF3D7_0631500-4R | TCTATCTTCCAATGAATTATGATG |
| PF3D7_0701500-1F | TGGAAAATGAGATGGAAAATC |
| PF3D7_0701500-1R | TCTGATGGGTCTGTTAATGTATAG |
| PF3D7_0701500-2F | GTTTTATCAACCATGACATTAGC |
| PF3D7_0701500-2R | TTCTGTGTGTATATTTTGTCCTTC |
| PF3D7_1039800-1F | GTTTTATCAACCATGACATTAGC |

| | |
|---|---|
| PF3D7_1039800-1R | TGGAAAATGAGATGGAAAATC |
| PF3D7_1039800-2F | TTCTGTGTGTATATTTTGTCCTTC |
| PF3D7_1039800-2R | TCTGATGGGTCTGTTAATGTATAG |
| PF3D7_0823400-1F | CTGCAAGTAGTTGTCAATTAGAAG |
| PF3D7_0823400-1R | ATCTTGAGATTCATTCCAACC |
| PF3D7_0823400-2F | TCAGAATCTTCAACGCAAAC |
| PF3D7_0823400-2R | AAATAATTTCCAATAAGCATATACATC |
| PF3D7_1029500-1F | AAAAGAACAAGATGTTGATGAAA |
| PF3D7_1029500-1R | TTTGGAAGGGCAGATAAAAT |
| PF3D7_0315900-1F | TATGTATCCTTCTAAATAAACAATGG |
| PF3D7_0315900-1R | AAATATGATTGTTAGACGTTAATGC |
| PF3D7_0315900-2F | AGGTTTAATGAAATAACACATTGC |
| PF3D7_0315900-2R | TTTGGTGGTTTCTTATATGTCTTC |
| PF3D7_1464200-1F | TCATAAACAAAATTGTATCTTTTCTC |
| PF3D7_1464200-1R | AAATTTACATGCAGGATTATGAC |
| PF3D7_1464200-2F | GGGGATAACAAATTAGAAAATAATG |
| PF3D7_1464200-2R | CTGCGTATAATCTTCTTCTTCAC |
| PF3D7_1465800-1F | CATTTACAAATTTATTGGCTAGG |
| PF3D7_1465800-1R | TTTTCATCAGATTTATTTATCGAAC |
| PF3D7_1465800-2F | TAAAATGTCTAAGTGTGGGTTTG |
| PF3D7_1465800-2R | TTAATTCGTTCTTTTAAATCTTCG |
| PF3D7_0408100-1F | TCAAGAGAAAGTTATATGCGTTG |
| PF3D7_0408100-1R | TGTTCAATCCTTTGTTCTTGTAG |
| PF3D7_0408100-2F | AAACGAAGAGAAACCTATAGACG |
| PF3D7_0408100-2R | CTTTGAGTTCTTGAATAATTTCG |
| PF3D7_1441600-1F | AAATATGCTATAATGAAATCAGGAG |
| PF3D7_1441600-1R | ACATGGTTGTTATTTGTTGTTTG |
| PF3D7_1441600-2F | TTAATTTCAGCATCCGATTTAG |
| PF3D7_1441600-2R | CATCATTTAAAGAATCGGGATAG |
| PF3D7_0112100-1F | GAAATTGAAAATATAGCAACACG |
| PF3D7_0112100-1R | TATAAGACTTTGCTTAAACGACTG |
| PF3D7_0112100-2F | AATGAGAAAATTGTTAAGTGGTG |
| PF3D7_0112100-2R | TCCCATTTTGTAGAATCTTTATG |
| PF3D7_1473700-1F | ATTTGTATTGAATGTTTGAATCG |
| PF3D7_1473700-1R | ATATCATTTTCTCTTGCACAATC |
| PF3D7_1473700-2F | CAAAATCCACTACAACTATCACG |
| PF3D7_1473700-2R | GGTGTACATAACAAAATTCATCC |
| PF3D7_0309700-1F | ATTTATTGCTCCGAATATTGAAC |
| PF3D7_0309700-1R | CATTTAGCAATTATTTTCCCTAAC |
| PF3D7_0309700-2F | TTGAAAGATAAAAGAGTTTCTTCG |
| PF3D7_0309700-2R | TATTATGCTCGTTCCTTTCTATG |
| PF3D7_0501700-1F | GACTTAACAATAATCGCCAAAG |
| PF3D7_0501700-1R | GTTTATTTTCCCATTCTTCTATTAAC |
| PF3D7_0501700-2F | TTGTAAGCAATGATAAAGATTCAG |
| PF3D7_0501700-2R | TACAACAATTCCATCTTCATTTAC |
| PF3D7_0221500-1F | AACCTTGACCCTCAAACTTC |

| | |
|---|---|
| PF3D7_0221500-1R | TCACTATTTATGTATTGTAATGTGAGAC |
| PF3D7_1237400-1F | AATATACGTTTCATACCTTTTCATC |
| PF3D7_1237400-1R | AATTAGGTAACTTCGAACAATCA |
| PF3D7_0425100-2F | AACTTGTATTGGAAGCCTTTG |
| PF3D7_0425100-2R | TAAAGAACCACAAAGTGCTAGAG |
| PF3D7_1400900-3F | AGTAAAATGCGTAGAACATTCAG |
| PF3D7_1400900-3R | TTCTTCTAGCGTAAATTGTTTTG |
| PF3D7_1400900-1F | ACCGTACATGCTATAATTCAATG |
| PF3D7_1400900-1R | ATGGATGCTCCATCTTTAACTA |
| PF3D7_1400900-2F | TGATTTATCAAAACAATTTACGC |
| PF3D7_1400900-2R | CCAAAACAAGATTCCATATACC |
| PF3D7_1478300-1F | TACATTGGTTCCGGTATTTG |
| PF3D7_1478300-1R | TTTCATTATATACTTCTTTGTGTTGC |
| PF3D7_1478300-2F | GGGTCATATGTATCAACATTTACAG |
| PF3D7_1478300-2R | AACATGCAAATACCGGAAC |
| PF3D7_1479300-1F | AATTTATCTGAAGTAAAATGCGTAG |
| PF3D7_1479300-1R | ATTATAACCTTCTGAACTGAAAGC |
| PF3D7_1479300-2F | TGATTTATCAAAACAATTTACGC |
| PF3D7_1479300-2R | ACCCAAAACAAGATTCCATATAC |
| PF3D7_0221200-1F | GAGCTGGTGCAGTTATAAGTATG |
| PF3D7_0221200-1R | GAAAAGACAACGAACAAAACTAC |
| PF3D7_0221200-2F | AATTCCTTAAAATCAACTTCTGG |
| PF3D7_0221200-2R | AACTGCACCAGCTCCTAATAC |
| PF3D7_0222200-1F | GAAAATGAGATGGAAAATCATATAG |
| PF3D7_0222200-1R | TCTGATGGGTCTGTTAATGTATAG |
| PF3D7_0222200-2F | GTTTTATCAACCATGACATTAGC |
| PF3D7_0222200-2R | TTCTGTGTGTATATTTTGTCCTTC |
| PF3D7_1303200.1-1F | ATAATACCACCACATACATCCAG |
| PF3D7_1303200.1-1R | AAATGCATAGGGAGTAAGAAAAC |
| PF3D7_1303200.1-2F | TAAGGAATTGGGTTTTCTTACTC |
| PF3D7_1303200.1-2R | AAACCCTCATATTTTCTTTCAATAC |
| PF3D7_1359100-1F | TTAACTTTAGATATTTTGTCATGGAG |
| PF3D7_1359100-1R | TACTTACTTCCTCTTCCAAATCC |
| PF3D7_1359100-2F | AAGGTAATGAACACGACAAAAG |
| PF3D7_1359100-2R | TCATCAGACATGATTAAATTGTTG |
| PF3D7_0618900.1-1F | CAAAAGAATAATATGAACGATGG |
| PF3D7_0618900.1-1R | TTTTGATTTATCGACATTTTGC |
| PF3D7_0618900.1-2F | AATGATTGTGATAAGGAACAATTAG |
| PF3D7_0618900.1-2R | TTTCATAATTTTCTAGGCATTTG |
| PF3D7_0615700-1F | TTTGTAAAGTTGTAAATGCTTGG |
| PF3D7_0615700-1R | GATAATCCTTCAATTTCTGAACC |
| PF3D7_0615700-2F | AGGTTCCTACGGAAGTAATTATG |
| PF3D7_0615700-2R | TACCATTTGATCTATCTGTGTGG |
| PF3D7_0604700-1F | CTCTTGGAAATTTAATATTGTGTTC |
| PF3D7_0604700-1R | TTATATCCGTCTAGGTCTTTTACC |
| PF3D7_0604700-2F | GGGCTTGGAGTACACTTAAAA |

| | |
|---|---|
| PF3D7_0604700-2R | ATCTTCGTCTGGTAATATACACG |
| PF3D7_0605900-1F | TCCTCCCAGGATTTTATGTC |
| PF3D7_0605900-1R | ATTTCCGAACAAAATTAATAAGG |
| PF3D7_0605900-2F | GTAGATTTTGTTGACACCATTTT |
| PF3D7_0605900-2R | ACTTGTATTCACCCACATGATTA |
| PF3D7_0609100-1F | CAAAACATAAGCAGAAAAGTACG |
| PF3D7_0609100-1R | TATATCACCATGTGTATGAGGTG |
| PF3D7_0609100-2F | TGATTATTGGAACATCAACTGAC |
| PF3D7_0609100-2R | AAAGATAAAGAAACCGTAACACC |
| PF3D7_0819900-1F | AATGGAAAGAAATTTAGACATGG |
| PF3D7_0819900-1R | TCATAGCAGATGATACTAGGATAATAGA |
| PF3D7_0819900-2F | AATATGATTTTATCCAATGTACGTG |
| PF3D7_0819900-2R | TTGACTGTTTCTTCATCATTTTC |
| PF3D7_1005300-1F | AGGAATGTTGAGAATGAAAAGAC |
| PF3D7_1005300-1R | TCCTTGCATAATTCTTAAAACG |
| PF3D7_1005300-2F | GTTGCCTATTTGACATACTTCTG |
| PF3D7_1005300-2R | TTTTCGTCTTTTCATTCTCAAC |
| PF3D7_1005300-3F | AAGAATTATTTCATTCAGCATATTTT |
| PF3D7_1005300-3R | TATGCTTCTTTTAATGGGAATG |
| PF3D7_1337100-1F | AATCTTTGTGATGAAAATTATGG |
| PF3D7_1337100-1R | ATATTCGAAGGCCAAATATAAAC |
| PF3D7_0615900-1F | ATCCAAACAAACAGAAAAGAGAC |
| PF3D7_0615900-1R | TCTATACAATCGTTTTGTTGTCC |
| PF3D7_0615900-2F | TATTACATCAAAGGAATGTTTCG |
| PF3D7_0615900-2R | TTAACCAAATTGTGGGAATAAG |
| PF3D7_0713200-4F | TGATGGAAGGACAAAATATACG |
| PF3D7_0713200-4R | CAATATCATTTTCTTTTAATATCTCG |
| PF3D7_0713200-1F | TTTTGAGACAATGTAAAAGTTGC |
| PF3D7_0713200-1R | TTCAATACATAATTAGATGAGGACAC |
| PF3D7_0604300-1F | AATAATGTTCCCTGGTCATCTAC |
| PF3D7_0604300-1R | TTATTCATGATTTTGTCATTGTG |
| PF3D7_0604300-2F | TACATCCACAACAAAGGAAATC |
| PF3D7_0604300-2R | CATTCTGTTACGAGTATTGTTCC |
| PF3D7_1442800-1F | CACAACATGAAAATGTAATAGGC |
| PF3D7_1442800-1R | CATCTTCCCTTTGTATATCCTTT |
| PF3D7_1442800-2F | TAAACTTCTATCGAACAGGAAGA |
| PF3D7_1442800-2R | AAAATGTAAATGCGAATCCTT |
| PF3D7_0727800-1F | TTTGTTATAAGCATACGGAGAAG |
| PF3D7_0727800-1R | AAACCTTTTCATTATTTCAGGAG |
| PF3D7_0727800-2F | ATCTTTTCGTTGTGTATAACAGG |
| PF3D7_0727800-2R | CCTACAAAATATATGCACACCTC |
| PF3D7_1459300-1F | AAAAGAAATAGTTTAAAAGAAGCAAA |
| PF3D7_1459300-1R | ATTACGAATATTTTGTTTTGTGG |
| PF3D7_1459300-2F | TGAGAACAAATCAGTAAATGTAGGT |
| PF3D7_1459300-2R | TTTTTTGCTTCTTTTAAACTATTTC |
| PF3D7_0909900-1F | AAATGCATATCCTCCTATTGAAC |

| | |
|---|---|
| PF3D7_0909900-1R | AATAACATAACCCTTCCGATAAC |
| PF3D7_0909900-2F | ATACCACTCATCATTCGATACAG |
| PF3D7_0909900-2R | ATACCTGCATTATTTACATTTGG |
| PF3D7_1476100-1F | GAAAACTATTTGGGATGAAGTTG |
| PF3D7_1476100-1R | TTTTGTATTTCTTCTTGAGATGG |
| PF3D7_1476100-2F | ATAGCCATTTTAAGGAAAAGAAG |
| PF3D7_1476100-2R | CAATATCTTCAATTTCGTTTTTG |
| PF3D7_0823100-1F | GAGTAAGTGAAGAAGAATTTGAGG |
| PF3D7_0823100-1R | CTCTCTTCCTGTTGGATTTTC |
| PF3D7_0823100_2F | ATATTAGATCCTGAATTTGATGAAG |
| PF3D7_0823100_2R | AATAATTCTTCATTACACCACTTTG |
| PF3D7_1145000-1F | ATTATGTAAACCTGGATCTGTTG |
| PF3D7_1145000-1R | TTCAGTGTAGTCATGTGTTTCAG |
| PF3D7_1145000-2F | TTGAAAATTCTGAAACACATGAC |
| PF3D7_1145000-2R | TCAATTCTTGACCTTGTATAGTTG |
| PF3D7_1109400-1F | AATTTAGCGAATGAACAAAATG |
| PF3D7_1109400-1R | TTCTTCAATAATGTCATCAAAGTG |
| PF3D7_1109400-2F | AAAACACAAGCACTTGAGGAC |
| PF3D7_1109400-2R | ATGCATTTCATTGTTCTTTATTC |
| PF3D7_1429400-1F | TTCCTCCTGAACTCTATGAAATC |
| PF3D7_1429400-1R | TTCATATTTGTCTTCTCTTGTCG |
| PF3D7_1429400-2F | ATTCACATATTTACAACGTAGGG |
| PF3D7_1429400-2R | TTATCATTCGATATGCTCTCTTG |
| PF3D7_1416200-1F | ATAGTGAACATGGAGGAGATAGC |
| PF3D7_1416200-1R | AGTGTTGCAATATAAAGGTGATG |
| PF3D7_1416200-2F | CTTATTGTTTATGTGCAGCTACC |
| PF3D7_1416200-2R | TTCACCTGTTTTATAGGATTCAG |
| PF3D7_0935200-1F | CCAATAGGATATGAAACCTTACC |
| PF3D7_0935200-1R | AAAGCTCTTATGGATGCTATTTC |
| PF3D7_0935200-2F | AAATCATGGCCTCTAATTTTAAC |
| PF3D7_0935200-2R | GACTACTGTTGTGTCGTTTGC |
| PF3D7_1108900-1F | AATAATAATTGTGATGGCATGTG |
| PF3D7_1108900-1R | TCTGTGTTGTTGGTCTCTTTTAC |
| PF3D7_1108900-2F | TTGAGTTTCCAGATGATACATTC |
| PF3D7_1108900-2R | TGATATTTGCTACATCATTTTCG |
| PF3D7_1225000-1F | CATAAATCAACATGTACCAAACC |
| PF3D7_1225000-1R | TTTAAAGCATAATTGCTCTTGTC |
| PF3D7_1225000-2F | ATTTATACCAACAATTTTCTTACTTACA |
| PF3D7_1225000-2R | AGTGACATGTTCCTACCCTTTA |
| PF3D7_0717600-1F | TTCGGAGAATTGTAATTATGAAG |
| PF3D7_0717600-1R | TACCTATATGAAGAACAGCATGG |
| PF3D7_0717600-2F | GGAGTAGCTATAGGTGAAATTGG |
| PF3D7_0717600-2R | AATCATTACAAGTCTCTTCATCG |
| PF3D7_1334100-1F | ATGAATAGTTCCAACTCAACTCC |
| PF3D7_1334100-1R | AGTCTTTCTTCTTGATCGTTCTC |
| PF3D7_1334100-2F | AAAATATGACACAACATTCGTTC |

| | |
|---|---|
| PF3D7_1334100-2R | CATCTTCTTGTGGTTTGTGATAG |
| PF3D7_0617100-1F | CCAGAAAGTACAAACTTATGCAG |
| PF3D7_0617100-1R | AAATATCCAAAAGAACCTATGATG |
| PF3D7_0617100-2F | TCGATCCATATATACAAAATGAAG |
| PF3D7_0617100-2R | TAGACTTGTGCGTTATTTCAATC |
| PF3D7_0821600-1F | AACAAACGAATAGGTTATCTGAAC |
| PF3D7_0821600-1R | AAAACCATCTTCACCCTTATTC |
| PF3D7_0821600-2F | ACGTGCAGGGTATTAAAGAAG |
| PF3D7_0821600-2R | CTTGATGTTGTTCAGTATTATTTCC |
| PF3D7_1304600-1F | TTATCTATCGATTCTGAAATGGTC |
| PF3D7_1304600-1R | TTATTGTGTAGAAATTTTAAGTCGTC |
| PF3D7_1304600-2F | TAACCACAACCAACCTTTAATAC |
| PF3D7_1304600-2R | GGTGATTTCTTCACCTTCTTC |
| PF3D7_1212200-1F | GAGTAGGAAAGAACAAGAAATGG |
| PF3D7_1212200-1R | TCTTCATTATGTTCAAAGGTTTC |
| PF3D7_1212200-2F | CGAATGTGAAATTAATGGTAAAC |
| PF3D7_1212200-2R | ACATACATATTCTTTGGATTCCTC |
| PF3D7_1113000-1F | CAACATATTTGTGATTATGTACCAC |
| PF3D7_1113000-1R | AATATTTATTTTCGTTTGGTTCC |
| PF3D7_1113000-2F | AATTGATGTAATTCCTAAAAGCAC |
| PF3D7_1113000-2R | CTATTTCGTAAAAGAAAATCATAGC |
| PF3D7_1221600-1F | ATCAACAAGTACTGATCATTTGC |
| PF3D7_1221600-1R | TTGCTCCAACATCCTTTATATACT |
| PF3D7_1221600-2F | AACCCTAGTTGTGAAAATGAAAT |
| PF3D7_1221600-2R | CATATCAAATGTTCTGATTCTCC |
| PF3D7_0417900-1F | AGGGAAGAAGTACGTTAGAAGG |
| PF3D7_0417900-1R | ATTTGTTTCATTTGTTTCATTTG |
| PF3D7_0417900-2F | CATATTGTAAAAGGAATGAAGAATG |
| PF3D7_0417900-2R | TCCTCATCATCTATTTTGTTGTG |
| PF3D7_1107700-1F | AAAGAAAACTTACCTGTACACCTG |
| PF3D7_1107700-1R | TTGATTGAGCATTTTATTTATGG |
| PF3D7_1107700-2F | AACAACCCACATGAACATACTAC |
| PF3D7_1107700-2R | ACATGTCCATTATCAGCTTCAC |
| PF3D7_0936900-1F | ACGTTTGGAATTAGATTTGTATG |
| PF3D7_0936900-1R | CATTTCTCATTATTTCTGACCAC |
| PF3D7_0936900-2F | ATGAAAGTCATGAAAGTTCAATC |
| PF3D7_0936900-2R | ATTCACGTTCTCCAGTTTTAAG |
| PF3D7_1314800-1F | AAAATTGAAGAAGAAAATCAAATG |
| PF3D7_1314800-1R | TATCAGGTATGTCCCTTTTATCC |
| PF3D7_1314800-2F | TTATATCAAGGGAAAAATGGACT |
| PF3D7_1314800-2R | AGATAAATCAAATAAGGCAAAGG |
| PF3D7_0217000-1F | TAAACAAAAGGAGTTTTCCAAAG |
| PF3D7_0217000-1R | GTAGGAGTCGAGATAAGAAGAAAG |
| PF3D7_0217000-2F | CAATGCTTATTCTTCATATATCTCAC |
| PF3D7_0217000-2R | AAAATAAAAGAATGTAAACTAAAATGG |
| PF3D7_1301500-1F | CCGATATGTCAAAGAATGATATG |

| | |
|---|---|
| PF3D7_1301500-1R | TTCTTTTGTTAATGGTTTCGTAG |
| PF3D7_1301500-2F | AAGTGTAGCAAAGGAAGGTTTAG |
| PF3D7_1301500-2R | TCATTAATTTCGTCCATAAAATC |
| PF3D7_0502100-1F | AATTGATGTGTATTCGTCTAATTATC |
| PF3D7_0502100-1R | CATTTGACCATTTTGTATTAACC |
| PF3D7_0502100-2F | GTGACGAGGAAAATGAAGAAG |
| PF3D7_0502100-2R | CAAGATCATTTGATAATTCTGGA |
| PF3D7_1337700-1F | CCACAAAATGCATCTATCATATC |
| PF3D7_1337700-1R | TGTAAATCATCCTCATCTTCAAC |
| PF3D7_1337700-2F | TGAATGTAATACCTATGCGTGAG |
| PF3D7_1337700-2R | ACATAATTGCATTTGTTGATAGG |
| PF3D7_1133100-1F | TGATAGTGACGACGAAATTTATG |
| PF3D7_1133100-1R | TGAATCACTGGTAGTTTTAGGATAG |
| PF3D7_1133100-2F | AAGTATTAAAGAGGGAATTCTTGAC |
| PF3D7_1133100-2R | ATTATTGTTGTTGTTGTTGTTGC |
| PF3D7_0912800-1F | AATTCTTGTAGCTCCATCATACC |
| PF3D7_0912800-1R | TCGTAGTAACTTCAACCATATCC |
| PF3D7_0912800-2F | CATGAAATTATTGAACCTAAGATATTG |
| PF3D7_0912800-2R | TAGGACTTGTTAGTTCATGTTCG |
| PF3D7_0928400-1F | GAATGTAATTCTTTTCCTGTGC |
| PF3D7_0928400-1R | ACCCAGCATCATTCGTTATAG |
| PF3D7_0928400-2F | TTGGTTATACGGTTACTATTGATG |
| PF3D7_0928400-2R | CTTATAATGGTTGGTATGGTTTTAC |
| PF3D7_0905300-1F | TTGATGGTTCTAAAATTATGACG |
| PF3D7_0905300-1R | CAAGATATTTCAGTTTCACTTGC |
| PF3D7_0905300-2F | GAGCATCAGACAAATAAAACAAG |
| PF3D7_0905300-2R | CATCTTCTCATCTTCAAATTCAC |
| PF3D7_0524100-1F | TGAAGTGGAGAAGCAAAATATC |
| PF3D7_0524100-1R | TTGTCTTTTACGTTTTGACAATC |
| PF3D7_0524100-2F | AATGATGTTACAAATTTTAGGTACG |
| PF3D7_05241002R | AATATTCCCTTCAAAGATCTCG |
| PF3D7_1346200-1F | AATAACATGGAATGTACCAAAAG |
| PF3D7_1346200-1R | CAAATAATTGGTAATCAAGAATCC |
| PF3D7_1346200-2F | AGCTAAGGAAAATGGAAGTATAGA |
| PF3D7_1346200-2R | TTTTAATTTTTTGTTTCCCTACA |
| PF3D7_1405300-1F | TTCAACATATCAATTTCAATTCC |
| PF3D7_1405300-1R | CAACAAGTGAGTTCTTATACAAATG |
| PF3D7_1405300-2F | TCTGAGAAGGAAAATGAAAGC |
| PF3D7_1405300-2R | TCGCACTTTGTATTATATGTTGG |
| PF3D7_0916600-1F | CTATCCTTGATATTGGATGTGG |
| PF3D7_0916600-1R | CAAAGATTGTTTTGGCTAGTTC |
| PF3D7_0916600-2F | GGTTTCTTCTGAATTGCATAAAT |
| PF3D7_0916600-2R | ACAATTTTCTTCAAACCATTCTT |
| PF3D7_0901200-1F | TTTAGTTAAAGATGGAGCATCAA |
| PF3D7_0901200-1R | TTTCTAATCTTTTGGGATTTTTC |
| PF3D7_1475400-1F | TAATTATGAGAGCTACAGCATCG |

| | |
|---|---|
| PF3D7_1475400-1R | TTTCATTTGAAGTTTTCATTTTG |
| PF3D7_1475400-3F | AGAATATGAGCGATCAGGTG |
| PF3D7_1475400-3R | TGTTCATCATACCAAATAGAAGC |
| PF3D7_1475400-3F | AATTAGCCGCAGTTTTGG |
| PF3D7_1475400-3R | TGATGTTTGAATCTTCCTTTTG |
| PF3D7_0532100-1F | TAATATCCTCTATTGCCACAGG |
| PF3D7_0532100-1R | CTTCAACATCTGAATCTAAATCG |
| PF3D7_1301100-2F | AAGTAAAATGCGTAGAACATTCA |
| PF3D7_1301100-2R | TTTTCTTAGTTCCAACACCATTA |
| PF3D7_1301100-3F | ATATGTGACAAAAGTGAAACAGAG |
| PF3D7_1301100-3R | CTTAATCCTGAATGTTCTACGC |
| PF3D7_0208500-1F | AAAGATGGAGTGTCCTTACAAAT |
| PF3D7_0208500-1R | TTTTTAAAGAGCTAGATGGGTTT |
| PF3D7_0211400-1F | AAAAGGGATGAAAATATATCAATG |
| PF3D7_0211400-1R | TGATAACCATGTCTATATCTGAGG |
| PF3D7_0615100-1F | AGTCGAAAAGGTTATTTAGATGC |
| PF3D7_0615100-1R | TAATGAAATAATGCTGGATTGAG |
| PF3D7_0615100-2F | TGCTGGTATAGGAGATACAAATG |
| PF3D7_0615100-2R | ATTATAAACAGGAGGCCAAATAC |
| PF3D7_0501300-1F | AGAAGCTTTACTTAATGAATACGAAG |
| PF3D7_0501300-1R | CAAAGAATAGGAATATCACAACATAG |
| PF3D7_0501300-2F | TGTAATACCAGAAAATACTGAATCC |
| PF3D7_0501300-2R | TGAGCCATTCTACGTTGC |
| PF3D7_1133400-1F | TGCAGATATTCCTGAACATAAAC |
| PF3D7_1133400-1R | AATAGTTGCTAATACAGCGACAG |
| PF3D7_1133400-2F | GAACGCTAGTATGATCAAAAGTG |
| PF3D7_1133400-2R | CCAATTATAACCCTTACCATGAC |
| PF3D7_1335900-1F | ATTCCATATTCACCATTATCTCC |
| PF3D7_1335900-1R | TTTTCATTATTTCTACCATGTGG |
| PF3D7_1335900-2F | TGGACTTGCTTATAAATTCGTAG |
| PF3D7_1335900-2R | TTCACCTAATGTTTCATCAAAAG |
| PF3D7_0709900-1F | ATATTTTACATCAGCCACCTATG |
| PF3D7_0709900-1R | GACATCTACATCTAGCCAATCAC |
| PF3D7_0709900-2F | CCTTTCATGATATCGTAAGTAGC |
| PF3D7_0709900-2R | CGCTTAAATAATACTCAGGGTTC |
| PF3D7_1143500-1F | CATCTCTCAAGCAATTTTCATAC |
| PF3D7_1143500-1R | AATTTTCTGTGTTTTCGTCATAG |
| PF3D7_1143500-2F | AAAATATGTTGTGGATTCTCTTTC |
| PF3D7_1143500-2R | TGTTTATTTGAATATGTGCCTATG |
| PF3D7_0625000.1-1F | TCCTACATGTCAACCATTAAAAC |
| PF3D7_0625000.1-1R | GGTACATTCATAAACTTGAGCAG |
| PF3D7_0625000.1-2F | CATTATTTTGAAGGACAAGAAAAC |
| PF3D7_0625000.1-2R | TATTATGGTAGCACAAACCAAAC |
| PF3D7_0625100-1F | CAACATTAGAAACGTGCTTACC |
| PF3D7_0625100-1R | GAATGTCCTGATACAATCAAATC |
| PF3D7_0625100-2F | AATATTCATGCAAAGCCATTC |

| | |
|---|---|
| PF3D7_0625100-2R | GCATAATCTAAGGTATAATTCAATCC |
| PF3D7_1147200-1F | TCAAATATTCCAAATTGTCAAAG |
| PF3D7_1147200-1R | AACTACTTCTCTCCACCTTATGC |
| PF3D7_1147200-2F | GGAATTTTCACTTTATTCAGAGG |
| PF3D7_1147200-2R | TTACTACCCATACCTTCATTTCC |
| PF3D7_1471500-1F | TTTTGGATATTTTGTGATACTGC |
| PF3D7_1471500-1R | TTTGAGTTCCTTATAATCCTTTTC |
| PF3D7_1471500-2F | TTGTACAGGGGCATATGAAG |
| PF3D7_1471500-2R | TAAGTGAAAGGGTATAGGAAACG |
| PF3D7_0801900-1F | GATGACGCTGAAGAATATTATGA |
| PF3D7_0801900-1R | ATTATTTTCCTTTCTGGGTGTAA |
| PF3D7_0801900-2F | ATGTACAAACAAAACGTATGACC |
| PF3D7_0801900-2R | TTTTGTATAATTTCCACATGCTC |
| PF3D7_0315600-1F | CTTGCAACTGCACGTATAATC |
| PF3D7_0315600-1R | TGCATAAATCGTTTTCTTTATTG |
| PF3D7_0315600-2F | CCGAGAATAATTTTACCTCACC |
| PF3D7_0315600-2R | TTGCGTTGTCTTTTGTTTG |
| PF3D7_0513700-1F | ATATGTGCAACTCAATGACAAAG |
| PF3D7_0513700-1R | TTCTGAAGAAAATGTAATTCTGTATG |
| PF3D7_0513700-2F | TTTCATAAAGGTGGCCATAAC |
| PF3D7_0513700-2R | TTGAATATCCATAGGAACAGATATTAG |
| PF3D7_1462600-1F | TTGTTTTCGTATGGACAAATG |
| PF3D7_1462600-1R | TTGAAGTACAAATAGAACGAAATG |
| PF3D7_1462600-2F | ATTATCCTCTGATGCTCCTTTAG |
| PF3D7_1462600-2R | GTTTCAAGGAAGGTTTGTCTATC |
| PF3D7_1215200-1F | CGTATGATTGAAAATGTAGGAAC |
| PF3D7_1215200-1R | AGTAAAATTGAAGAATGGAGGTC |
| PF3D7_1215200-2F | TGTAGGAACAAAATTAGGACATC |
| PF3D7_1215200-2R | TTCAGTAAAATTGAAGAATGGAG |
| PF3D7_1121500-1F | AAATATAGGCCATCTGTTGTTTC |
| PF3D7_1121500-1R | GGAATATCGTTTATTAATTCCATC |
| PF3D7_1121500-2F | AAAGAACAACCGTATTAGAAGAAG |
| PF3D7_1121500-2R | AAGCCTCCTCTTTATCACTCTC |
| PF3D7_1129600-1F | ATATAATCAAACGACAGGTTGTG |
| PF3D7_1129600-1R | GCTACTTATTTTGTGATGCTCTG |
| PF3D7_1129600-2F | GTACGAGTGATCATATTTCAACC |
| PF3D7_1129600-2R | ATTCTTGTGCGTTTTATCTTTTC |
| PF3D7_1301700-1F | CGTTACAGATTACAACAACACCT |
| PF3D7_1301700-1R | GCTGCTCTAACATTATTGAAATG |
| PF3D7_0220200-1F | TTATTTTCTGGTGTAGATGATGG |
| PF3D7_0220200-1R | ATTGATTTTCACCTTCTTTCATT |
