# Supplementary material for: Identification of Novel Pre-Erythrocytic Malaria Antigen Candidates for Combination Vaccines with Circumsporozoite Protein
Source: PLoS One. 2016 Jul 19;11(7):e0159449. doi: 10.1371/journal.pone.0159449 (PMC4951032; doi:10.1371/journal.pone.0159449)
Supplement: S2 Table — Table shows the list of primer used for generation of vaccine construct. Start codon in forward primer and stop codon in reverse primer are shown in lower case letter. (PDF) [file pone.0159449.s007.pdf]

S2 Table. Primers to amplify inserts for DNA vaccine constructs

Py DNA vaccines	
**Gene Name**	**Primer sequence with LIC appendage**
PyCSP - F	CGC CCA GCG GCA CCG GC atg TTAAACGAGCTATGTTACAATGA
PyCSP - R	CAC GCA CGC GAG CGG GC tta ACATTTATCCATTTTACAAATTTCAG
PyPF3D7_1308500 - F	CG CCC AGC GGC ACC GGC atg ATAATAAGAGCAGATATTGAAAGGAG
PyPF3D7_1308500 - R	CA CGC ACG CGA GCG GGC tta GTATATTATATTAAGGGCCTGCTC
PyPF3D7_0727200 - F	CG CCC AGC GGC ACC GGC atg GTCTTTAAAATTTCCAAAACTCTG
PyPF3D7_0727200 - R	CA CGC ACG CGA GCG GGC tta TGGATAATTTCCAGATGTATTTTT
PyPF3D7_0730200 - F	CG CCC AGC GGC ACC GGC atg AGCGAGATTAACAATTTAAAAGAA
PyPF3D7_0730200 - R	CA CGC ACG CGA GCG GGC tta AAAATCTATGAATATGTCCCTGAA
PyPF3D7_0818900 - F	CG CCC AGC GGC ACC GGC atg GCTAACGCAAAAGC
PyPF3D7_0818900 - R	CA CGC ACG CGA GCG GGC tta ATCAACTTCTTCAACAGTTGGTC
PyPF3D7_1026400 - F	CG CCC AGC GGC ACC GGC atg ACATCATTAAAATGGAATATATTTGG
PyPF3D7_1026400 - R	CA CGC ACG CGA GCG GGC tta TCTGATTATTTCATAACAATTTTCA
PyPF3D7_1111200 - F	CG CCC AGC GGC ACC GGC atg GAAACGGGGGTCAATATTAGCA
PyPF3D7_1111200 - R	CA CGC ACG CGA GCG GGC tta CTCAAATTGGGCACTATTACGTTTC
PyPF3D7_1122200 - F	CG CCC AGC GGC ACC GGC atg AGGAATAATATAGAAAATGTAAA
PyPF3D7_1122200 - R	CA CGC ACG CGA GCG GGC tta TTTCTCATTTGAAAAGGATTTG
PyPF3D7_1134000 - F	CG CCC AGC GGC ACC GGC atg GCTGGCTTTAATAAAAATG
PyPF3D7_1134000 - R	CA CGC ACG CGA GCG GGC tta TGCATTATCTTTGTTTTCTTCT
PySLARP1 - 4F	CG CCC AGC GGC ACC GGC atg AACGATTTTAATTTGGGCTTAA
PySLARP1 - 4R	CA CGC ACG CGA GCG GGC tta GCTTTGATTAGTATCACAACCACT
PySLARP1 - 6F	CGC CCA GCG GCA CCG GC atg TATTTACGAGGAAGACAAGTTCA
PySLARP1 - 6R	CAC GCA CGC GAG CGG GC tta CATTATTTCAAAATTTATATGGGAGTT
PyPF3D7_1302200 - F	CGC CCA GCG GCA CCG GC atg AACCCTTGCTTTTGTGAGGATGCAG
PyPF3D7_1302200 - R	CA CGC ACG CGA GCG GGC tta TTTTGGT TGATATTGTT CTTTAAGAAA
PyPF3D7_1323000 - F	CG CCC AGC GGC ACC GGC atg ATAATTTTTGTTTACGTCCTTTTTC
PyPF3D7_1323000 - R	CA CGC ACG CGA GCG GGC tta TTTTGACATTGCAAAAATCATAT

PyPF3D7_1411500 - F	CG CCC AGC GGC ACC GGC atg TTTTTCTCATATATAGGCACCAT
PyPF3D7_1411500 - R	CA CGC ACG CGA GCG GGC tta TTGTATATATTTCATGTTAAAATGTGG
PyLISP1 - 1F	CG CCC AGC GGC ACC GGC atg ATAGAATACAACTTACTAGGAAGTGCAC
PyLISP1 - 1R	CA CGC ACG CGA GCG GGC tta CCTCACCTTAAAATATGGATCACC
PyLISP1 - 2F	CG CCC AGC GGC ACC GGC atg GGTGATCCATATTTTAAGGTGAG
PyLISP1 - 2R	CA CGC ACG CGA GCG GGC tta CTGATCAATGGTAATAAACTCG
PyLISP1 - 3F	CG CCC AGC GGC ACC GGC atg GAGTTTATTACCATTGATCAGAATGG
PyLISP1 - 3R	CA CGC ACG CGA GCG GGC tta TGCATTTGTATGATGTAAAAGTAAATA
PyPF3D7_1434400 - F	CG CCC AGC GGC ACC GGC atg TATTATATTACACAAAAAATATTTCAG
PyPF3D7_1434400 - R	CA CGC ACG CGA GCG GGC tta ATAAAAATAATGAGAAGAATTCTTGG
PyPF3D7_1456100 - F	CG CCC AGC GGC ACC GGC atg AAAATTGTTAAAGAACTTTCTAAAA
PyPF3D7_1456100 - R	CA CGC ACG CGA GCG GGC tta TGATGGGGATGGAAACA
PyPF3D7_0103400 - 2F	CGC CCA GCG GCA CCG GC atg TTTAGGCTTTATAGTAGTGGA
PyPF3D7_0103400 - 2R	CAC GCA CGC GAG CGG GC tta AAGACATATCCCAAAACTAACCC
PyPF3D7_0103400 - 3F	CGC CCA GCG GCA CCG GC atg AACTTGAGTGGAAAATTAAATAATATG
PyPF3D7_0103400 - 3R	CAC GCA CGC GAG CGG GC tta CTTAGCATGCCAATTCG
PyPF3D7_0304300 - F	CG CCC AGC GGC ACC GGC atg ATTGGTAATATAAAAGGGAAACAA
PyPF3D7_0304300 - R	CA CGC ACG CGA GCG GGC tta AACGTCGTTAGTAATTTTGTCA
PyPF3D7_0405500 - F	CG CCC AGC GGC ACC GGC atg AACGAAAAAATTATTAAGGA
PyPF3D7_0405500 - R	CA CGC ACG CGA GCG GGC tta TTCTTTACTATCAACTTCTATTTTTTCT
PyPF3D7_0506200 - F	CG CCC AGC GGC ACC GGC atg GACGAAAACGATGATCT
PyPF3D7_0506200 - R	CA CGC ACG CGA GCG GGC tta GTAAATATCTTGAAAAACGGTGTTA
PyPF3D7_1207400 - F	CG CCC AGC GGC ACC GGC atg AGTTATTCCAATAGTAGCATAAAA
PyPF3D7_1207400 - R	CA CGC ACG CGA GCG GGC tta CGCCAAATTTATGGAATGA
PyPF3D7_1241500 - F	CG CCC AGC GGC ACC GGC atg GAAAAACACCTTGAAAAA
PyPF3D7_1241500 - R	CA CGC ACG CGA GCG GGC tta TGCATGAAATATAGATGGTG
***Pb* DNA vaccines**	
**Gene Name**	**Primer sequence with LIC appendage**
PbCSP - F	CGC CCA GCG GCA CCG GC atg GGATATGGACAAAATAAAAGCAT

PbCSP - R	CAC GCA CGC GAG CGG GC tta TATACTTGAACATTTATCCATTTTACAA
PbPF3D7_1308500 - F	CG CCC AGC GGC ACC GGC atg TTATTAAGAAAGAGATGCATTGAAA
PbPF3D7_1308500 - R	CA CGC ACG CGA GCG GGC tta GTTAGATGAGTAAATTATATTAAGAGCC
PbPF3D7_0727200 - F	CG CCC AGC GGC ACC GGC atg AAAGTCTTTAAAATTGCAAA
PbPF3D7_0727200 - R	CA CGC ACG CGA GCG GGC tta GGTCCATATAAATTTGGGGTA
PbPF3D7_0730200 - F	CG CCC AGC GGC ACC GGC atg AGCGAGATTAACAATTTAAAAGAAG
PbPF3D7_0730200 - R	CA CGC ACG CGA GCG GGC tta CATAAAATCTATGAATATGTCCCTG
PbPF3D7_0818900 - F	CG CCC AGC GGC ACC GGC atg GCTAACGCAAAAGC
PbPF3D7_0818900 - R	CA CGC ACG CGA GCG GGC tta ATCAACTTCTTCAACAGTTG
PbPF3D7_1026400 - F	CG CCC AGC GGC ACC GGC atg TTTTGTGAGTTTATAACAAATG
PbPF3D7_1026400 - R	CA CGC ACG CGA GCG GGC tta TTCATAATAATTTTCAAAGGGAAA
PbPF3D7_1111200 - F	CG CCC AGC GGC ACC GGC atg GAATATAAATCCTTATTAATTGAAAAAC
PbPF3D7_1111200 - R	CA CGC ACG CGA GCG GGC tta GAAACTTAATTTCTCAGCTATATTTTT
PbPF3D7_1122200 - F	CG CCC AGC GGC ACC GGC atg AGGAATAGTATAGAAAATGTAAATG
PbPF3D7_1122200 - R	CA CGC ACG CGA GCG GGC tta AAAAGAAGAATTAAAGACCCTTAAC
PbPF3D7_1134000 - F	CG CCC AGC GGC ACC GGC atg TTTGAGGTAAAAGCTACAAATG
PbPF3D7_1134000 - R	CA CGC ACG CGA GCG GGC tta GCTTTCTTGAACCATTTT
PbSLARP - F	CG CCC AGC GGC ACC GGC atg AATGAAGTAAAATATAATGAAGGCT
PbSLARP - R	CA CGC ACG CGA GCG GGC tta GGAGCTCCTTAATGGATATAGAAA
PbPF3D7_1302200 - F	CG CCC AGC GGC ACC GGC atg AACACCCTCAATGTCTTT
PbPF3D7_1302200 - R	CA CGC ACG CGA GCG GGC tta TTTAAGAAAATGCTCCACACC
PbPF3D7_1323000 - F	CG CCC AGC GGC ACC GGC atg AAACTTTTTATAATTTTTGTTTACG
PbPF3D7_1323000 - R	CA CGC ACG CGA GCG GGC tta TTTTGACATTGCAAAAATCA
PbPF3D7_1411500 - F	CG CCC AGC GGC ACC GGC atg AAGGAAAATTGTACGAAGAAAAG
PbPF3D7_1411500 - R	CA CGC ACG CGA GCG GGC tta TTCTAAAAATTTATGAAAACATTG
PbLISP1 - 1F	CG CCC AGC GGC ACC GGC atg AAAAATAGAGAATACAACTTACTAGGAAA
PbLISP1 - 1R	CA CGC ACG CGA GCG GGC tta CTCACAATTGCAGTTATTACCAT
PbLISP1 - 5F	CG CCC AGC GGC ACC GGC atg GGTAATATGAGCCATTTTGAATT
PbLISP1 - 5R	CA CGC ACG CGA GCG GGC tta GAATATGGAAATAGAAAAATCAAT
PbPF3D7_1434400 - F	CG CCC AGC GGC ACC GGC atg GATAATATTGGTGTTTCTGAAGAAG

PbPF3D7_1434400 - F	CA CGC ACG CGA GCG GGC tta AAAATAATGGGAAGAATTTTTGG
PbPF3D7_1456100 - F	CG CCC AGC GGC ACC GGC atg GAACTCTCTAAAAACGGGAAA
PbPF3D7_1456100 - R	CA CGC ACG CGA GCG GGC tta TGATGGGGATGGAAAC
PbPF3D7_0103400 - 1F	CG CCC AGC GGC ACC GGC atg CTCCAAGGGAAAGAAAAAAT
PbPF3D7_0103400 - 1R	CA CGC ACG CGA GCG GGC tta CATTGCATAACTTGCATTCGTTT
PbPF3D7_0103400 - 2F	CG CCC AGC GGC ACC GGC atg GAAACGAATGCAAGTTATGC
PbPF3D7_0103400 - 2R	CA CGC ACG CGA GCG GGC tta TCTACCACTCTCTGTCTTTTTCTTA
PbPF3D7_0304300 - 1F	CG CCC AGC GGC ACC GGC atg CTAAAGTTTTCGAAATATTTTTA
PbPF3D7_0304300 - 1R	CA CGC ACG CGA GCG GGC tta TACGATTTCATTGCAAAATAGG
PbPF3D7_0304300 - 2F	CG CCC AGC GGC ACC GGC atg CTATTTTGCAATGAAATCGTACT
PbPF3D7_0304300 - 2R	CA CGC ACG CGA GCG GGC att ACGCGTTTTACAAAAAAAAA
PbPF3D7_0405500 - F	CG CCC AGC GGC ACC GGC atg AACGAAAAAATTATTAAGGAC
PbPF3D7_0405500 - R	CA CGC ACG CGA GCG GGC tta TATTTCTTTACTATTAACTTCTATCTTTT
PbPF3D7_0506200 - F	CG CCC AGC GGC ACC GGC atg CTTTTAAACAAAAATAATACATTGG
PbPF3D7_0506200 - R	CA CGC ACG CGA GCGGGC tta TACTAGGACATTGTAAATATCTTGAAAAAC
PbPF3D7_1207400 - F	CG CCC AGC GGC ACC GGC atg GAATTAGAAGAAAAAGAAGACGAA
PbPF3D7_1207400 - R	CA CGC ACG CGA GCG GGC tta CGCCAAATTTATGGAATGA
PbPF3D7_1241500 - F	CG CCC AGC GGC ACC GGC atg GAAAAACAACTTGAAAAGG
PbPF3D7_1241500 - R	CA CGC ACG CGA GCG GGC tta CTTTTTATTTTCTTCAAATGTATCA
