# Supplementary material for: Identification of Novel Pre-Erythrocytic Malaria Antigen Candidates for Combination Vaccines with Circumsporozoite Protein
Source: PLoS One. 2016 Jul 19;11(7):e0159449. doi: 10.1371/journal.pone.0159449 (PMC4951032; doi:10.1371/journal.pone.0159449)
Supplement: S3 Table — Table shows the list of predicted peptides from indicated antigens. (PDF) [file pone.0159449.s008.pdf]

S3 Table. Peptides used for in vitro stimulation of splenocytes from DNA immunized mice

PyCSP	PyPF3D7_1456100	PySLARP	PyPF3D7_1207400	PyPF3D7_0506200
SYVPSAEQI	SANSNLTFL	HYQMMINNI	KYILACNSI	SSANLDTEI
	FNYEFITTL	AQYNYLNDL	LYIKSINNI	KLNTVFQDI
	IMIITFTLL	NYQITNMPL	TYMSPINQI	IYNVLVQYK
	QSLQNYNSL	YYVNDNDHI	VVFEKDTFL	KNFKIENII
	SNLTFLEYL	INFNSLNHI	VSKTKLIRL	LVYRYKPTS
	LYIKNNDKI	NYIHQDSSN	CYNYKCDAV	KSFDKNNQI
	FYRKGFKQI	SQNSQNSNI	DTFLRYLYI	KSLQKLNTV
	ICYEYEDNL	IYDNNRQNI		SNLKSVILI
	ININKSTYV	RTFCNYNNI		
	TNYLTALGM	VYTYRNNNI		
	STYKQKSYL	KYTKGIQTT		
		LYRNNNKYI		
		SIQNTQNSI		
