# Supplementary material for: Identification of Novel Pre-Erythrocytic Malaria Antigen Candidates for Combination Vaccines with Circumsporozoite Protein
Source: PLoS One. 2016 Jul 19;11(7):e0159449. doi: 10.1371/journal.pone.0159449 (PMC4951032; doi:10.1371/journal.pone.0159449)
Supplement: S4 Table — Table shows the primer sequence used to generate recombination construct for myc-tagging of selected Py protein. Gene ID of selected Py protein is shown in the primer name. (PDF) [file pone.0159449.s009.pdf]

S4 Table. Primers to amplify construct inserts for myc-epitope tagging of proteins in transgenic *Py* parasites

Primer Name	Primer Sequence
SacII-PyPF3D7_0730200rep FW	CCGCGGCGGCGGAATCCCCTTA
SpeI-PyPF3D7_0730200rep-REV	ACTAGTCATAAAATCTATGAATATGTCCC
SacII-PyPF3D7_1241500-5U-FW	AAACCGCGGAGCACTAGCTGGTATAGTGCCTATAAG
XbaI-PyPF3D7_1241500-REV	GGGTCTAGATGCATGAAATATAGATGGTGTTGC
SacII-PyPF3D7_0506200-5U-FW	AAACCGCGGTATTGGCTCGTTTCGAATTTTTTCAAACTC
XbaI-PyPF3D7_0506200-REV	GGGTCTAGAACTTTTATATTGTACTAAGACA
