# Supplementary material for: Identification of Novel Pre-Erythrocytic Malaria Antigen Candidates for Combination Vaccines with Circumsporozoite Protein
Source: PLoS One. 2016 Jul 19;11(7):e0159449. doi: 10.1371/journal.pone.0159449 (PMC4951032; doi:10.1371/journal.pone.0159449)
Supplement: S5 Table — LS burden in mice vaccinated with the indicated antigen was compared to that in mice vaccinated with EV. Antigens are listed from greatest to smallest reduction in LS burden. Only antigens that significantly reduced LS burden are shown. CSP is shown in bold. (PDF) [file pone.0159449.s010.pdf]

S5 Table. LS burden reduction after vaccination with individual antigens.

***Pb* single antigen**

Antigen	% Reduction	P-value (N)
**CSP[a]**	**84.36**	**<0.001 (53)**
PF3D7_0730200[a]	74.82	0.019 (5)
PF3D7_0818900[a]	68.63	0.019 (5)
PF3D7_1411500[a]	61.18	0.008 (10)
**CSP[b]**	**59.96**	**<0.001 (19)**
LISP1-1[a]	58.96	0.018 (5)
PF3D7_1111200[a]	57.49	0.018 (5)
LISP1-1[b]	55.59	0.033 (5)
PF3D7_1456100[b]	54.28	0.015 (4)
LISP1-5[b]	53.95	0.010 (5)
PF3D7_1456100[a]	51.35	<0.000 (14)
LISP1-5[a]	50.84	0.023 (10)
PF3D7_1122200[a]	48.34	0.072 (5)[#]
PF3D7_1207400[b]	48.05	0.011 (5)
PF3D7_1241500[a]	44.42	0.001 (15)
**CSP[c]**	**41.46**	**0.006 (5)**
PF3D7_0506200[a]	34.33	0.005 (20)
PF3D7_1323000[b]	30.91	0.011 (5)
PF3D7_1241500[b]	26.48	0.015 (4)
PF3D7_0405500[a]	25.87	0.027 (10)
PF3D7_1111200[b]	24.84	0.015 (4)

***Py* single antigen**

Antigen	% Reduction	P-value
PF3D7_1323000[b]	90.22	0.001 (8)
**CSP[b]**	**76.23**	**<0.001 (33)**
PF3D7_1308500[c]	71.48	0.006 (5)
PF3D7_0727200[b]	70.48	0.001 (9)
PF3D7_1122200[b]	65.58	0.085 (9)[#]
PF3D7_1302200[b]	61.13	0.019 (10)
PF3D7_1434400[b]	60.96	0.021 (15)
PF3D7_1207400[c]	60.24	0.006 (5)
SLARP-4[c]	54.08	0.012 (5)
PF3D7_1122200[c]	52.84	0.006 (5)
PF3D7_0304300[c]	51.79	0.011 (5)
PF3D7_1411500[a]	50.65	0.011 (5)
LISP1-3[c]	48.11	0.006 (5)
PF3D7_1308500[a]	46.19	0.004 (10)
**CSP[a]**	**45.75**	**<0.001 (35)**
PF3D7_1241500[c]	45.42	0.006 (5)
PF3D7_1134000[a]	41.83	0.015 (10)
PF3D7_0730200[a]	40.99	0.009 (15)
PF3D7_0103400-2[a]	39.89	0.030 (5)
PF3D7_0103400-3[c]	39.73	0.006 (5)
PF3D7_1323000[c]	38.06	0.030 (5)
PF3D7_1122200[a]	35.88	0.006 (10)
**CSP[c]**	**34.17**	**0.001 (30)**
PF3D7_1026400[b]	33.17	0.047 (9)
PF3D7_1302200[a]	31.81	0.007 (10)
PF3D7_1241500[a]	30.30	0.013 (10)
PF3D7_1323000[a]	23.24	0.009 (10)
PF3D7_0730200[b]	19.57	0.056 (5)[#]
PF3D7_0304300[a]	17.12	0.047 (10)

Median percent reduction in LS burden per tested antigen, as compared to empty vector. Antigens are listed from greatest to smallest percent reduction. Only antigens that significantly (# or nearly significantly) reduced LS burden are shown. CSP is shown in bold.

[a]GG immunization; [b]EP immunization; [c]IM immunization. (N), Number of mice used for immunization
