# Supplementary material for: Identification of Novel Pre-Erythrocytic Malaria Antigen Candidates for Combination Vaccines with Circumsporozoite Protein
Source: PLoS One. 2016 Jul 19;11(7):e0159449. doi: 10.1371/journal.pone.0159449 (PMC4951032; doi:10.1371/journal.pone.0159449)
Supplement: S6 Table — LS burden in mice vaccinated with a combination of CSP and the indicated antigen, was compared to that in mice vaccinated with CSP in combination with EV. Antigens are listed from greatest to smallest reduction in LS burden. Only antigens that significantly reduced LS parasite burden are shown. Doubling the CSP dose did not significantly reduce LS parasite burden beyond that achieved with CSP in combination with EV. (PDF) [file pone.0159449.s011.pdf]

S6 Table. LS burden reduction after vaccination with CSP combination antigens.

Combination with *Pb* CSP		
**Antigen**	**% Reduction**	**P-value (N)**
LISP1-1[a]	79.08	0.047 (5)
SLARP-4[a]	76.07	0.047 (5)
PF3D7_1456100[a]	55.03	0.047 (5)
**2 x CSP[a]**	**53.48**	**0.081 (10)**

Combination with *Py* CSP		
**Antigen**	**% Reduction**	**P-value**
SLARP-4[b]	57.58	0.009 (10)
PF3D7_0506200[b]	57.41	0.093 (10)[#]
LISP1-3[b]	55.63	0.032 (10)
PF3D7_1207400[b]	49.10	0.081 (10)[#]
PF3D7_1411500[b]	40.85	0.061 (10)[#]
**2 x CSP[b]**	**39.96**	**0.154 (10)**
PF3D7_1241500[b]	27.42	0.061 (10)[#]

Median percent reduction in LS burden per tested combination vaccine of CSP and the indicated antigen, as compared to combined CSP and empty vector. Antigens are listed from greatest to smallest percent reduction. Only antigens that significantly (# or nearly significantly) reduced LS burden are shown. Double dose (2x) CSP is shown in bold. Doubling the CSP dose did not significantly reduce LS parasite burden beyond that achieved with CSP in combination with empty vector.

[a]GG immunization; [b]EP immunization; [c]IM immunization. (N), Number of mice used for immunization
